# Persistent Topological Laplacians—A Survey

**DOI:** 10.3390/math13020208

**Published:** 2025-01-09

**Authors:** Xiaoqi Wei, Guo-Wei Wei

**Affiliations:** 1 Department of Mathematics, Michigan State University, East Lansing, MI 48824, USA; 2 Department of Electrical and Computer Engineering, Michigan State University, East Lansing, MI 48824, USA; 3 Department of Biochemistry and Molecular Biology, Michigan State University, East Lansing, MI 48824, USA

**Keywords:** topological data analysis, topological Laplacians, persistent spectral theory

## Abstract

Persistent topological Laplacians constitute a new class of tools in topological data analysis (TDA). They are motivated by the necessity to address challenges encountered in persistent homology when handling complex data. These Laplacians combine multiscale analysis with topological techniques to characterize the topological and geometrical features of functions and data. Their kernels fully retrieve the topological invariants of corresponding persistent homology, while their non-harmonic spectra provide supplementary information. Persistent topological Laplacians have demonstrated superior performance over persistent homology in the analysis of large-scale protein engineering datasets. In this survey, we offer a pedagogical review of persistent topological Laplacians formulated in various mathematical settings, including simplicial complexes, path complexes, flag complexes, digraphs, hypergraphs, hyperdigraphs, cellular sheaves, and N-chain complexes.

## 1. Introduction

In recent years, there has been exponential growth in research on topological data analysis (TDA) in data science. TDA provides a set of mathematical and computational techniques for extracting insightful information from complex, high-dimensional datasets. The primary goal of TDA is to uncover and understand the underlying spatial features of data that might be difficult to capture using traditional methods from statistics, physics, and other mathematical disciplines. The main tools of TDA are adapted from homology theory. In homology theory, algebraic objects, such as groups, rings, or modules, are associated with geometrical objects to infer topological features from these algebraic representations. The most basic geometrical objects in homology theory are simplicial complexes, consisting of simplices that model various interactions in complex data. When the input is a point cloud, traditional (simplicial) homology theory only captures trivial topological information. Hence, it is impossible to deduce the shape of a point cloud solely through the calculation of simplicial homology. A major breakthrough in overcoming this limitation was the invention of persistent homology [1,2]. The basic idea is to construct a multiscale family of simplicial complexes, called a filtration, from the input point cloud and examine the evolution of these simplicial complexes and their associated homology groups across scales. The output of persistent homology consists of arrays of topological invariants computed on various scales, often visualized or represented by persistence diagrams, persistence barcodes [3], persistence images [4,5], or persistence landscapes [6]. Persistent homology has proven to be the most important technique in TDA.

Persistent homology has been applied in a wide variety of disciplines, including image processing [7,8], neuroscience [9], computational chemistry [10], computational biology [11–14], nanomaterials [15,16], crystalline materials [17], and complex networks [18], among others. One of the most remarkable applications of persistent homology is the dominant success of topological deep learning (TDL) models in the D3R Grand Challenges, a global competition series in computer-aided drug design [19,20]. Another notable achievement is the TDL-facilitated discovery of the evolutionary mechanism of SARS-CoV-2 [21]. The term “topological deep learning” was coined in 2017 [11] and has since become a trending topic in data science and machine learning [22]. The success of TDL, along with other topology-based machine learning algorithms, has made topological data analysis (TDA) a prominent subject in applied mathematics and data science.

However, since homology theory can only characterize topological spaces up to homotopy equivalence, persistent homology has many limitations when dealing with complex data. It neglects certain aspects of shape evolution in a filtration that might be important in applications. For example, in a filtration, the zeroth Betti number stops changing once all points are connected, even though the connectivity of simplicial complexes evolves. Additionally, for a heterogeneous 1-dimensional (1D) cycle, persistent homology does not account for the cycle’s composition or the number of points in the cycle. These features are particularly important for complex data, such as those encountered in biological sciences. Persistent Laplacians [23,24] address some of these challenges by introducing a multiscale version of combinatorial Laplacians. Roughly speaking, Laplacians are matrices whose spectra encapsulate both topological and non-topological information. A persistent Laplacian is defined on a pair of simplicial complexes, and its kernel is isomorphic to the corresponding persistent homology group. This means the harmonic spectra of persistent Laplacians can fully recover the barcodes output by persistent homology, while non-harmonic spectra capture additional information about the input filtration.

In spectral graph theory, the graph Laplacian, or Kirchhoff matrix, is extensively studied [25]. Given a graph, the number of zero eigenvalues of its graph Laplacian equals the number of connected components of the graph [26]. Beyond the number of connected components, many graph properties are related to the graph Laplacian, such as the relationship between the Fiedler value and graph connectivity [27]. However, the graph Laplacian only accounts for pairwise interactions. Indeed, the graph Laplacian can be seen as a special case, i.e., the first one, of a series of combinatorial Laplacians introduced by Eckmann in 1944 [28], which are defined for each dimension on a simplicial complex. It is well known that the kernel of a combinatorial Laplacian is isomorphic to the corresponding simplicial homology group [28].

The relationship between Laplacians and homology has been explored in many different contexts and domains. On a differentiable manifold, the de Rham-Hodge theory states that the kernel of a Hodge Laplacian is isomorphic to the corresponding de Rham cohomology group. The discretization of Hodge Laplacians can be achieved by the discrete exterior calculus [29,30] and the finite element exterior calculus [31]. The associated Helmholtz–Hodge decomposition has widespread applications in various fields [32]. Hodge Laplacians on graphs were discussed in [33]. The similarities and differences between combinatorial Laplacians on simplicial complexes and Hodge Laplacians on differentiable manifolds have been carefully examined [34]. These Laplacians have found applications in science and engineering, including ranking [35–37], graphics and imaging [36,38,39], games and traffic flows [40], deep learning [41], data representations [42], dimension reduction [43], denoising [44], object synchronization [45], link prediction [46], sensor network coverage [47], generalizing effective resistance to simplicial complexes [48], cryo-electron microscopy [49], brain networks [50], and biological interactions [51].

A persistent formulation of Hodge Laplacians on manifolds was introduced in 2019 [52]. The resulting evolutionary de Rham-Hodge theory can be viewed as persistent Hodge Laplacians [53]. Both persistent Hodge Laplacians and persistent (combinatorial) Laplacians are persistent topological Laplacians (PTLs) that extend the scope and capabilities of TDA. In the most general sense, any method that utilizes multiscale topological Laplacians to quantitatively characterize the topological/geometrical shapes of point cloud data or differentiable manifolds can be thought of as a persistent topological Laplacian approach.

Persistent Laplacians have been extensively studied in the past few years [54–56]. In addition to differential manifolds and simplicial complexes, persistent topological Laplacians have been formulated in many other mathematical settings, such as flag complexes [57], digraphs [58], cellular sheaves [59], hypergraphs [60], and hyperdigraphs [61]. Computational algorithms [56,62], including a software package [63], have been developed to compute persistent Laplacians. Persistent Laplacian approaches have been applied to protein-ligand binding prediction [64], interactomic network modeling [65], gene expression analysis [66], deep mutational scan [67], phylogenetic analysis[68], and SARS-CoV-2 variant analysis [69]. The advantage of persistent Laplacians over persistent homology was demonstrated with a collection of 34 datasets in protein engineering [70]. The power of persistent Laplacians has been exemplified by their successful prediction of the emerging dominant SARS-CoV-2 variants [71].

Both persistent homology and persistent topological Laplacians are constructed on the basis of the properties of chain complexes. It is possible to define a Mayer homology for the more general N-chain complexes [72], where N is an integer. Recently, Shen et al. introduced persistent Mayer homology and persistent Mayer Laplacians [73] to further extend persistent homology and persistent topological Laplacians to N-chain complexes, offering a new development in TDA.

Although there are numerous reviews and monographs on persistent homology [1,74–76], there is no review on persistent topological Laplacians. The primary goal of this survey is to introduce the notion of persistent topological Laplacians to a wider audience and facilitate further developments on the subject. In this survey, we will first introduce the basics of persistent homology and then discuss the theory of persistent Laplacians and some of their recent advances. The presentation of mathematics in this article is pedagogical, and we hope that the survey is accessible to researchers from diverse backgrounds.

## 2. Mathematical Preliminaries

### 2.1. Simplicial Complexes and Homology

**Figure 1.**
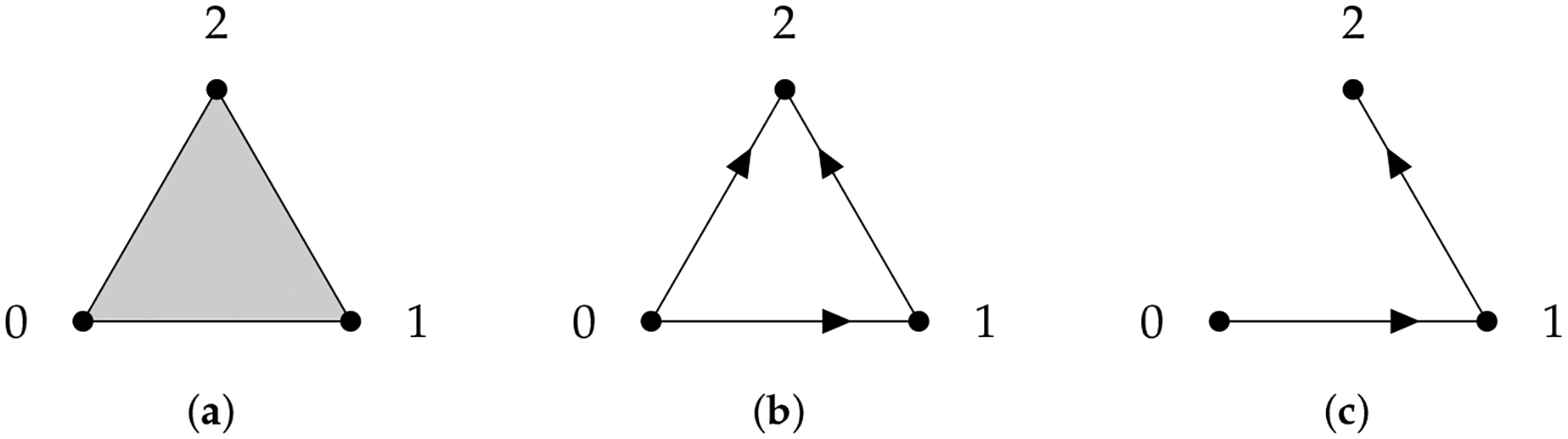
(**a**) The simplicial complex {{0}, {1}, {2}, {0, 1}, {0, 2}, {1, 2}, {0, 1, 2}}. (**b**) The simplicial complex {0, 1, 2, 01, 02, 12}. Arrows emphasize that vertices are ordered. (**c**) The simplicial complex {0, 1, 2, 01, 12}.

Given a finite set V, a simplicial complex X is a collection of subsets of V, such that if a set σ is in X, then any subset of σ is also in X. A set σ that consists of q+1 elements is referred to as a q-simplex. If σ is a subset of τ, then we say that σ is a face of τ and denote it by σ⩽τ. The definition of a simplicial complex may seem abstract, but it is closely related to geometry. A q-simplex can be realized as the convex hull of q+1 points in a real coordinate space, so it is possible to construct a polyhedron from a simplicial complex if the simplices are glued properly. For example, supposing X is the power set of {0, 1, 2}, we can identify X with a triangle whose vertices are labeled {0, 1, 2} (Figure 1a). However, many geometrical objects can be sliced properly so as to give rise to a simplicial complex. We always designate a fixed order of vertices in a simplicial complex (the choice of ordering will not affect the resulting homology groups [77]) and require that the vertices of any simplices should be ordered according to the fixed ordering. For example, suppose that we use the natural ordering 0 < 1 < 2 for the simplicial complex {{0}, {1}, {2}, {0,1}, {0,2}, {1,2}} (Figure 1b), then we must not write the simplex {0, 1} as {1, 0}. To emphasize that a simplex $\left\{v_{0},\ldots ,v_{q}\right\}$ is ordered, we will use the notation $\left[v_{0},\ldots ,v_{q}\right]$ or $v_{0}\ldots v_{q}$.

We now introduce a more abstract definition. A simplicial complex X gives rise to a sequence of vector spaces and linear maps, collectively referred to as a simplicial chain complex

$$\cdots \overset{\partial_{3}^{X}\;}{\to }C_{2}(X)\overset{\partial_{2}^{X}\;}{\to }C_{1}(X)\overset{\partial_{1}^{X}\;}{\to }C_{0}(X)⟶0.$$

The chain group $C_{q}(X)$ is the real vector space generated by q-simplices, and the boundary operator $\partial_{q}$ is a linear map such that

$$\partial_{q}\left[v_{a_{0}},\ldots ,v_{a_{q}}\right]=\sum_{i}(-1{)}^{i}\left[v_{a_{0}},\ldots ,{\overset{ˆ}{v}}_{a_{i}},\ldots ,v_{a_{q}}\right].$$

where the symbol ${\overset{ˆ}{v}}_{a_{i}}$ means that ${\overset{ˆ}{v}}_{a_{i}}$ is deleted. An element of $C_{q}(X)$ is called a q-chain and, by definition, is a linear combination of q-simplices. Sometimes, it is intuitive to regard a q-chain as a function mapping a q-simplex to its coefficient. The coefficients $(-1{)}^{i}$ ensure that $\partial_{q}\partial_{q+1}=0$, so the q-th homology group $H_{q}={\text{ker}}_{q}/\text{im}\partial_{q+1}$ is well-defined. The dimension of the homology group $H_{q}$ is referred to as the q-th Betti number, which is often described as counting the number of q-dimensional “holes” in a simplicial complex. It is not always clear what a high-dimensional hole represents in a simplicial complex; nevertheless, the main idea is that homology groups extract quantitative topological information about a simplicial complex.

**Example 1.** The simplicial complex X={0,1,2,01,02,12} (Figure 1b) *has only two chain groups*, $C_{0}$ *and* $C_{1}$, *and one boundary map $\partial_{1}$*, *represented by the matrix*

$$\begin{array}{cc} & \;01\;12\;02\\ \begin{array}{c} 0\\ 1\\ 2 \end{array} & \left(\begin{array}{ccc} -1 & 0 & -1\\ 1 & -1 & 0\\ 0 & 1 & 1 \end{array}\right) \end{array}$$

*if we identify any real-valued function $f_{1}:\{01,12,02\}\to R$ with the column vector* ${\left(f_{1}(01),f_{1}(12),f_{1}(02)\right)}^{T}$, *and any real valued function* $f_{0}:\{0,1,2\}\to R$ *with the column vector* ${\left(f_{0}(0),f_{0}(1),f_{0}(2)\right)}^{T}$. *We can see that $\partial_{1}f_{1}=f_{0}$ if and only if* $f_{0}(0)=-f_{1}(01)-f_{1}(02),f_{0}(1)=f_{1}(01)-f_{1}(12)$, *and* $f_{0}(2)=f_{1}(12)+f_{1}(02)$. *Since* $C_{2}=0$, *the homology group* $H_{1}$ *is ker $\partial_{1}$ and* $f_{1}\in H_{1}$ *implies* $f_{1}(01)=-f_{1}(02)=f_{1}(12)$.

*For the simplicial complex* Y={0,1,2,01,12} (Figure 1c), *the matrix representation of* $\partial_{1}$ is

$$\begin{array}{cc} & \;01\;12\\ \begin{array}{c} 0\\ 1\\ 2 \end{array} & \left(\begin{array}{cc} -1 & 0\\ 1 & -1\\ 0 & 1 \end{array}\right) \end{array}$$

*and we can verify that the only $f_{1}$ that satisfies* $\partial_{1}f_{1}=0$ *is the zero function. The intuition behind the difference between* $H_{1}(X)$ and $H_{1}(Y)$ is that in X the edges {01, 12, 02} constitute a close path, while in Y *there are no close paths*.

A simplicial chain complex is an example of a chain complex. The reader only needs to konw that a chain complex (V,d) is a sequence of vector spaces and linear morphisms

$$\cdots \overset{d_{3}\;}{\to }V_{2}\overset{d_{2}\;}{\to }V_{1}\overset{d_{1}\;}{\to }V_{0}⟶0$$

where $d_{q}d_{q+1}=0$. We often assume that each $V_{q}$ is a finite-dimensional inner product space.

### 2.2. Combinatorial Laplacians

Many simplicial complexes share the same Betti numbers. In this case, we can resort to a class of finer descriptors called combinatorial Laplacians to distinguish among different simplicial complexes. Before we define combinatorial Laplacians, we first need to equip a chain group with an inner product. The canonical approach is to let the set of q-simplices be an orthonormal basis for the q-th chain group $C_{q}$. Now we can discuss the adjoint of the boundary operator $\partial_{q}$, denoted by $\partial_{q}^{*}$, and the q-th combinatorial Laplacian $\Delta_{q}$ [28] is defined by

$$\partial_{q+1}\partial_{q+1}^{*}+\partial_{q}^{*}\partial_{q}.$$

When q=0, since $\partial_{0}=0$, the 0-th combinatorial Laplacian is just $\partial_{1}\partial_{1}^{*}$. The q-th combinatorial Laplacian is a positive semi-definite symmetric operator and only has non-negative eigenvalues. One fact of linear algebra is that, if U,V, and W are inner product spaces and f:U→V,g:V→W are two linear morphisms such that gf=0, then $\text{ker}\left(g^{*}g+ff^{*}\right)≅\text{ker}g/\text{im}f$. Therefore, the kernel of the q-th combinatorial Laplacian $\Delta_{q}$ is isomorphic to the q-th homology group $H_{q}$ [28]. This property guarantees that we can calculate Betti numbers from the spectra of combinatorial Laplacians. We can further show that $C_{q}$ admits a Hodge decomposition (a detailed exposition can be found in [33])

$$C_{q}=\text{im}\;\partial_{q}^{*}⊕\text{ker}\Delta_{q}⊕\text{im}\partial_{q+1}.$$

**Example 2.** *For a simple graph* (V,E), *let* $f_{0}$ be a function that maps every vertex to a real number. If we view the simple graph as a simplicial complex, then $\partial_{1}^{*}$ *maps* $f_{0}$ *to a real valued function whose domain is* E. *The Dirichlet energy of* $f_{0}$

$$\sum_{v_{i}v_{j}\in E}{\left|f_{0}\left(v_{i}\right)-f_{0}\left(v_{j}\right)\right|}^{2}=\left\langle \partial_{1}^{*}f_{0},\partial_{1}^{*}f_{0}\right\rangle =\left\langle f_{0},\partial_{1}\partial_{1}^{*}f_{0}\right\rangle$$

*measures how* $f_{0}$ *varies over* V. *Any* $f_{0}\in ker\;\Delta_{0}=ker\partial_{1}\partial_{1}^{*}$ *is a function with zero Dirichlet energy. In a connected graph, if $f_{0}$ has zero Dirichlet energy, then* $f_{0}(a)=f_{0}(b)$ *for any two vertices* a *and* $b\;(f_{0}$ *is a constant function*), *because there is always a path that starts from* a *and ends at* b. *If a graph has more than one connected components*, $f_{0}$ *only needs to be constant on any connected components. In other words, the dimension of ker* $\Delta_{0}$ *is equal to the number of connected subgraphs*.

**Figure 2.**
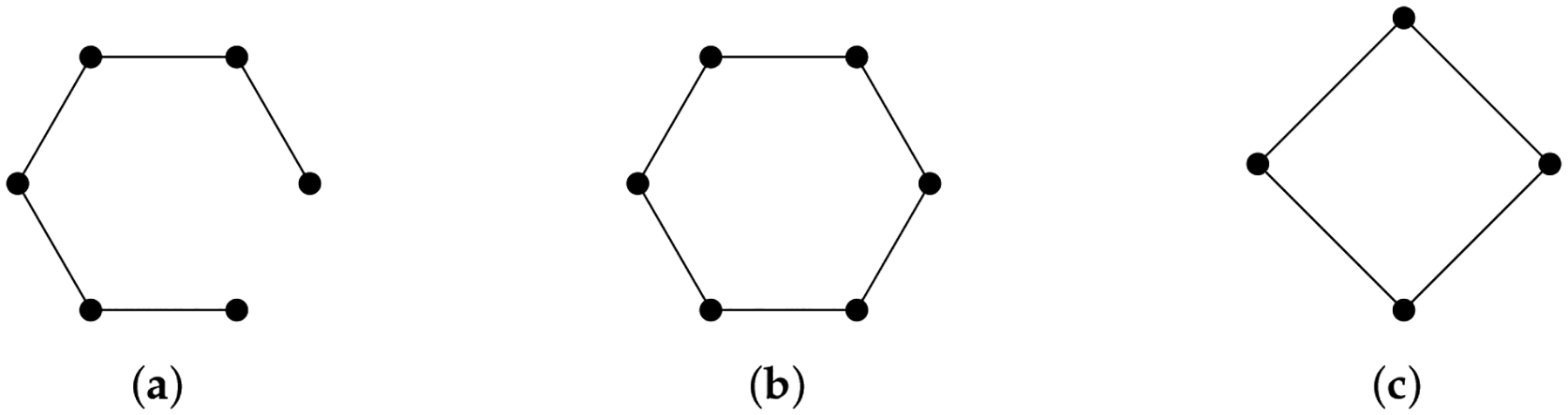
Homology can distinguish (**a**) from (**b**,**c**), but cannot distinguish between (**b**,**c**). Laplacians can distinguish among all of them. For a cycle graph with n vertices, the spectrum of the graph Laplacian is {2-2cos(2kπ/n)∣k=1,…,n}.

The operator $\Delta_{0}$ is more commonly known as the graph Laplacian, and there is extensive research studying the relationship between the spectrum of a graph Laplacian and properties of a graph [25]. For a connected graph, it is well known that the minimal nonzero eigenvalue of its graph Laplacian reflects the graph’s connectivity [78]. Graphs that share the same homology groups may have different graph Laplacians (Figure 2).

### 2.3. Filtration and Persistent Homology

So far we have introduced the elementary theory of simplicial complexes, but we have not explained how it is related to point-cloud data. A point cloud is a set of finitely many points $P=\left\{v_{0},\ldots ,v_{n}\right\}$ in a Euclidean space. Usually the geometrical structure of a point cloud is related to some non-geometrical properties of the object the point cloud represents, and a good understanding of the “geometry” of the point cloud is important. Here, the first problem is what we mean by “geometry”. A naive notion of “geometry” is the pairwise distances between each pair of points, and we can represent this information by a filtration, such that we obtain a nested sequence of simplicial complexes. One commonly used filtration is Vietoris-Rips filtration: given a point cloud $P=\left\{v_{0},\ldots ,v_{n}\right\}$ and a parameter $d\in R,\;X_{d}$ is a simplicial complex such that the simplex $\left\{v_{a_{0}},\ldots ,v_{a_{q}}\right\}\in X_{d}$ if and only if the Euclidean distance between $v_{a_{i}}$ and $v_{a_{j}}$ is at most d for any 0≤i<j≤q. Using d, we obtain finitely many distinct simplicial complexes, each of which characterizes the shape of the point cloud on a different scale. The homology groups or combinatorial Laplacians of each $X_{d}$ will change as d varies, providing a characterization of the point cloud.

**Figure 3.**
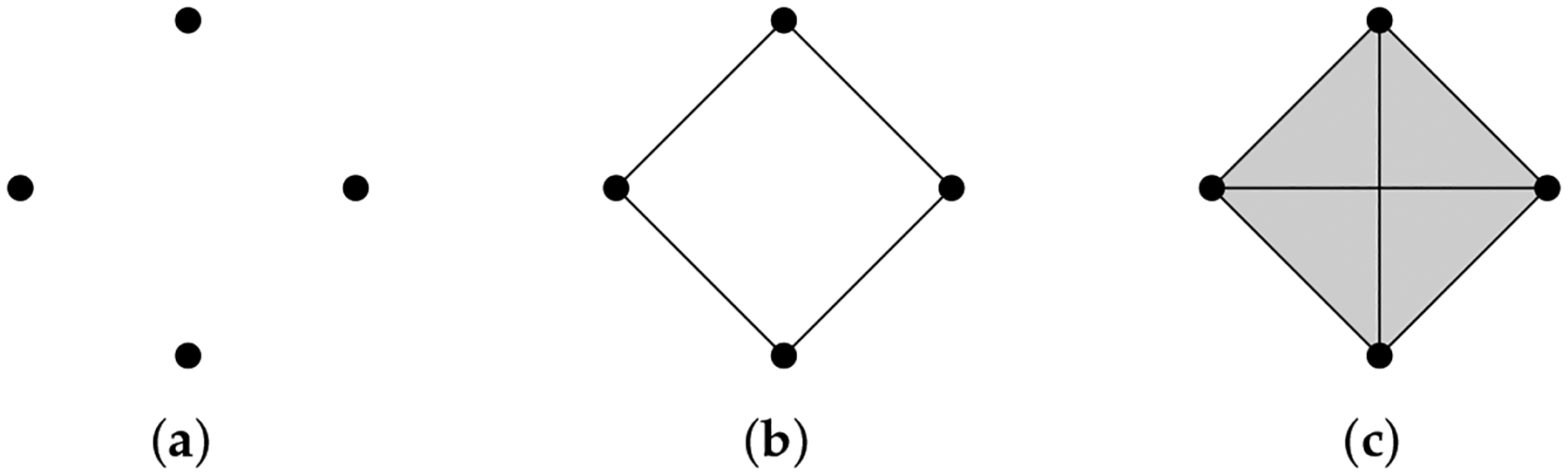
(**a**) $X_{0}=\{x,y,z,w\}$. (**b**) $X_{\sqrt{2}}=\{x,y,z,w,\text{xy},\text{yz},\text{zw},\text{xw}\}$. (**c**) $X_{2}=X_{\sqrt{2}}\cup \{\mathrm{xz},\mathrm{yw},\mathrm{yzw},\mathrm{xzw},\mathrm{xyw},\mathrm{xyz},\mathrm{xyzw}\}$

**Example 3.** *Now we build a Vietoris-Rips filtration from the point cloud* $\{x=(1,0),y=\;(0,1),z=(-1,0),w=(0,-1)\}\subset R^{2}$ *shown in* Figure 3a. *When* d=0, *there are no edges in* $X_{d}$. *When* $d=\sqrt{2},\;X_{d}$ *changes for the first time and becomes* {x,y,z,w,xy,yz,zw,xw}. *If* d *goes from* $\sqrt{2}$ *to* 2, $X_{2}=X_{\sqrt{2}}\cup \{\text{xz},\text{yw},\text{yzw},\text{xzw},\text{xyw},\text{xyz},\text{xyzw}\}$. *As d becomes bigger*, $X_{d}$ *contains more and more simplices. Let us examine* $H_{1}$ *for* $d=0,\;\sqrt{2},2.\;H_{1}\left(X_{0}\right)=0$ *as there is no close path, and $H_{1}\left(X_{\sqrt{2}}\right)=1$ because of four newly born edges. When* $d=2,\;H_{1}=0$ *since the close path is filled by high-dimensional simplices*.

In addition to calculating homology groups or combinatorial Laplacians for each $X_{t}$ in a filtration, we can also calculate the persistent homology to quantify how topological features of a smaller complex $X_{s}$ persist in $X_{t}$. Suppose X and Y are two simplicial complexes and X⊂Y, then we have the following diagram (dashed arrows indicates inclusion maps ι):


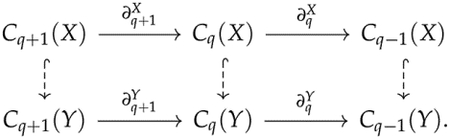




Since $\text{im}\;\partial_{q+1}^{Y}$ is larger than $\text{im}\;\partial_{q+1}^{X}$, some q-dimensional “holes” in X might be filled because of $\text{im}\;\partial_{q+1}^{Y}$. The q-dimensional “holes” in X that persists in Y are $\text{ker}\;\partial_{q}^{X}/\text{im}\;\partial_{q+1}^{Y}$, but since $\text{im}\;\partial_{q+1}^{Y}$ is not necessarily a subspace of $\text{ker}\;\partial_{q}^{X}$, the proper expression should be

$$\text{ker}\;\partial_{q}^{X}/\left(\text{im}\;\partial_{q+1}^{Y}\cap \text{ker}\;\partial_{q}^{X}\right)=\text{ker}\;\partial_{q}^{X}/\left(\text{im}\;\partial_{q+1}^{Y}\cap C_{q}(X)\right).$$

This quotient space is called the q-th persistent homology group of the pair X⊂Y, the dimension of which is referred to as the q-th persistent Betti number.

A more formal understanding of persistent homology is helpful. We notice that $\partial_{q}^{Y}\iota =\iota \partial_{q}^{X}$. In plain words, this means that the boundary of a simplex σ∈X is unchanged if we view it as a simplex in Y. In general, for two chain complexes $\left(V,d^{V}\right)$ and $\left(W,d^{W}\right)$, the collection of maps $f_{q}$ such that $f_{q+1}d_{q}^{V}=d_{q}^{W}f_{q}$ for all q is called a chain map. A chain map f induces a homomorphism $f_{•}:H_{q}(V)\to H_{q}(W)$, and sometimes the image $f_{•}\left(H_{q}(V)\right)$ is called the persistent homology group of the chain map $f:\left(V,d^{V}\right)\to \left(W,d^{W}\right)$.

## 3. Persistent (Combinatorial) Laplacians

### 3.1. Persistent Laplacians

We have shown that the kernel of the q-th combinatorial Laplacian is isomorphic to the q-th homology group. This result has been generalized for persistent homology groups.


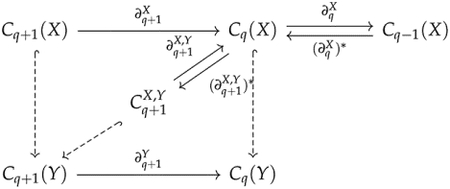




Given two complexes X⊂Y, let $C_{q+1}^{X,Y}$ be the subspace

$$\left\{c\in C_{q+1}(Y)|\partial_{q+1}^{Y}(c)\in C_{q}(X)\right\}$$

of $C_{q+1}(Y)$ and $\partial_{q+1}^{X,Y}:C_{q+1}^{X,Y}\to C_{q}(X)$ the restriction of $\partial_{q+1}^{Y}$ onto $C_{q+1}^{X,Y}$, then the persistent homology group $\text{ker}\;\partial_{q}^{X}/\left(\text{im}\;\partial_{q+1}^{Y}\cap C_{q}(X)\right)$ is equal to $\text{ker}\;\partial_{q}^{X}/\text{im}\;\partial_{q+1}^{X,Y}$. Since $C_{q+1}^{X,Y}$ inherits the inner product structure from $C_{q+1}(Y)$, and $\partial_{q}^{X}\partial_{q+1}^{X,Y}=0$, if we define the q-th persistent Laplacian $\Delta_{q}^{X,Y}:C_{q}(X)\to C_{q}(X)$ [23,24] by

(1) $$\partial_{q+1}^{X,Y}{\left(\partial_{q+1}^{X,Y}\right)}^{*}+{\left(\partial_{q}^{X}\right)}^{*}\partial_{q}^{X}$$

(where $\partial_{q+1}^{X,Y}{\left(\partial_{q+1}^{X,Y}\right)}^{*}$ is called the upper persistent Laplacian, denoted by $\Delta_{q,+}^{X,Y}$, and ${\left(\partial_{q}^{X}\right)}^{*}\partial_{q}^{X}$ the down persistent Laplacian, denoted by $\Delta_{q,-}^{X,Y}$), we can prove the persistent Hodge theorem

$$\text{ker}\;\Delta_{q}^{X,Y}≅\frac{\text{ker}\;\partial_{q}^{X}}{\text{im}\;\partial_{q+1}^{Y}\cap C_{q}(X)}$$

and the persistent Hodge decomposition

$$C_{q}(X)=\text{im}\;\partial_{q+1}^{X,Y}⊕\text{ker}\;\Delta_{q}^{X,Y}⊕\text{im}\;{\left(\partial_{q}^{X}\right)}^{*}$$

or equivalently

$$C_{q}(X)=\text{im}\;\Delta_{q,+}^{X,Y}⊕\text{ker}\;\Delta_{q}^{X,Y}⊕\text{im}\;\Delta_{q,-}^{X,Y}.$$

and the proofs are the same as those for combinatorial Laplacians. When $X=Y,\;\Delta_{q}^{X,Y}$ is just a combinatorial Laplacian. The persistent Hodge theorem implies that information of persistent Betti numbers is included in the spectra of persistent Laplacians, and additional information can be extracted from nonzero eigenvalues of persistent Laplacians. Given a point cloud and a filtration $\left\{X_{d},d\in R\right\}$ constructed from it, we can calculate persistent Laplacians for $X_{s}\leq X_{t}$ for a set of preselected s and t. Employing the information captured in non-harmonic spectra of persistent Laplacians can boost performance of persistent homology-based machine learning models. In fact, even the minimal nonzero eigenvalue of $\Delta^{X_{d},X_{d}}$ already provides a lot of extra information about the point cloud.

**Figure 4.**
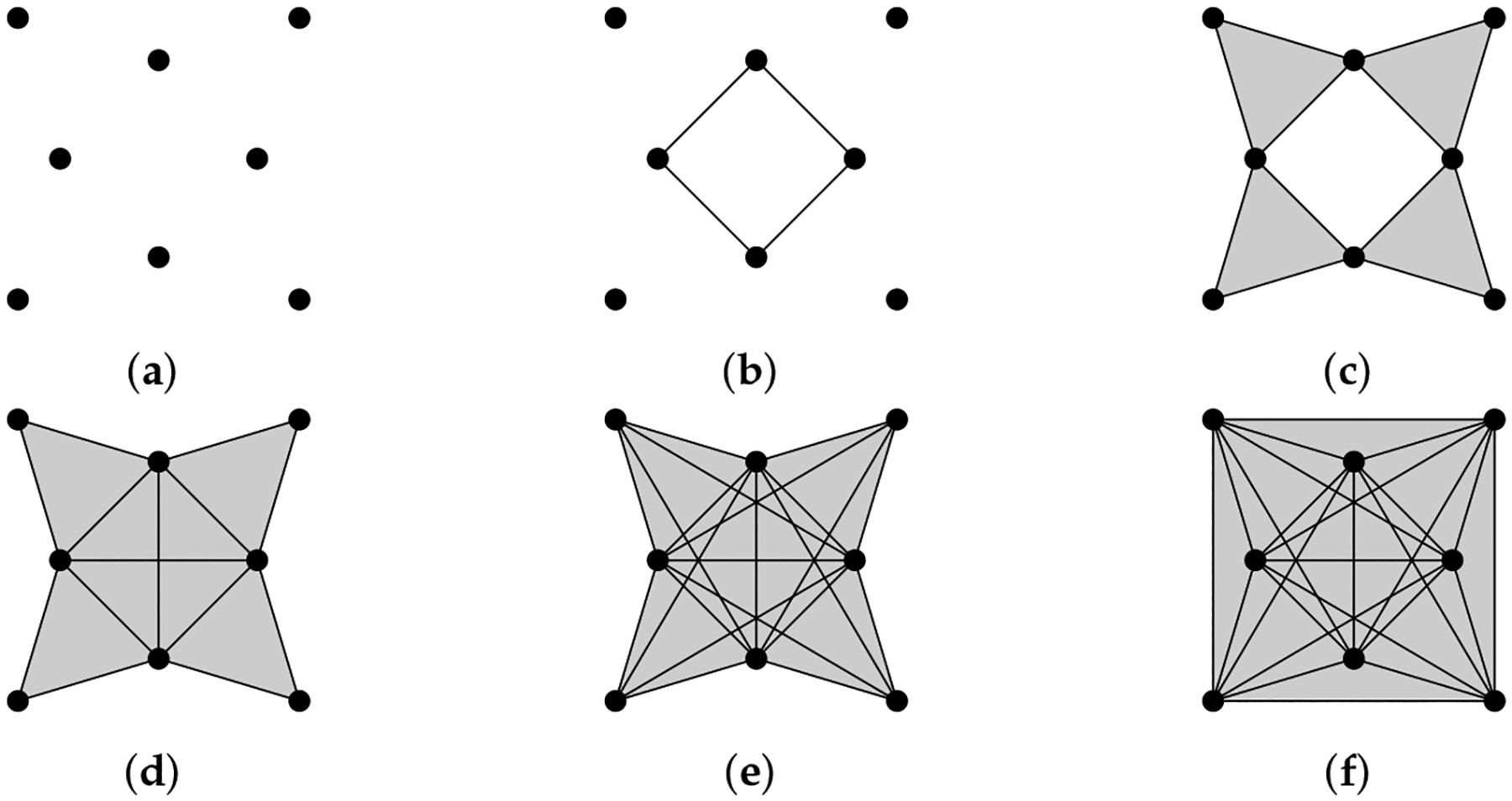

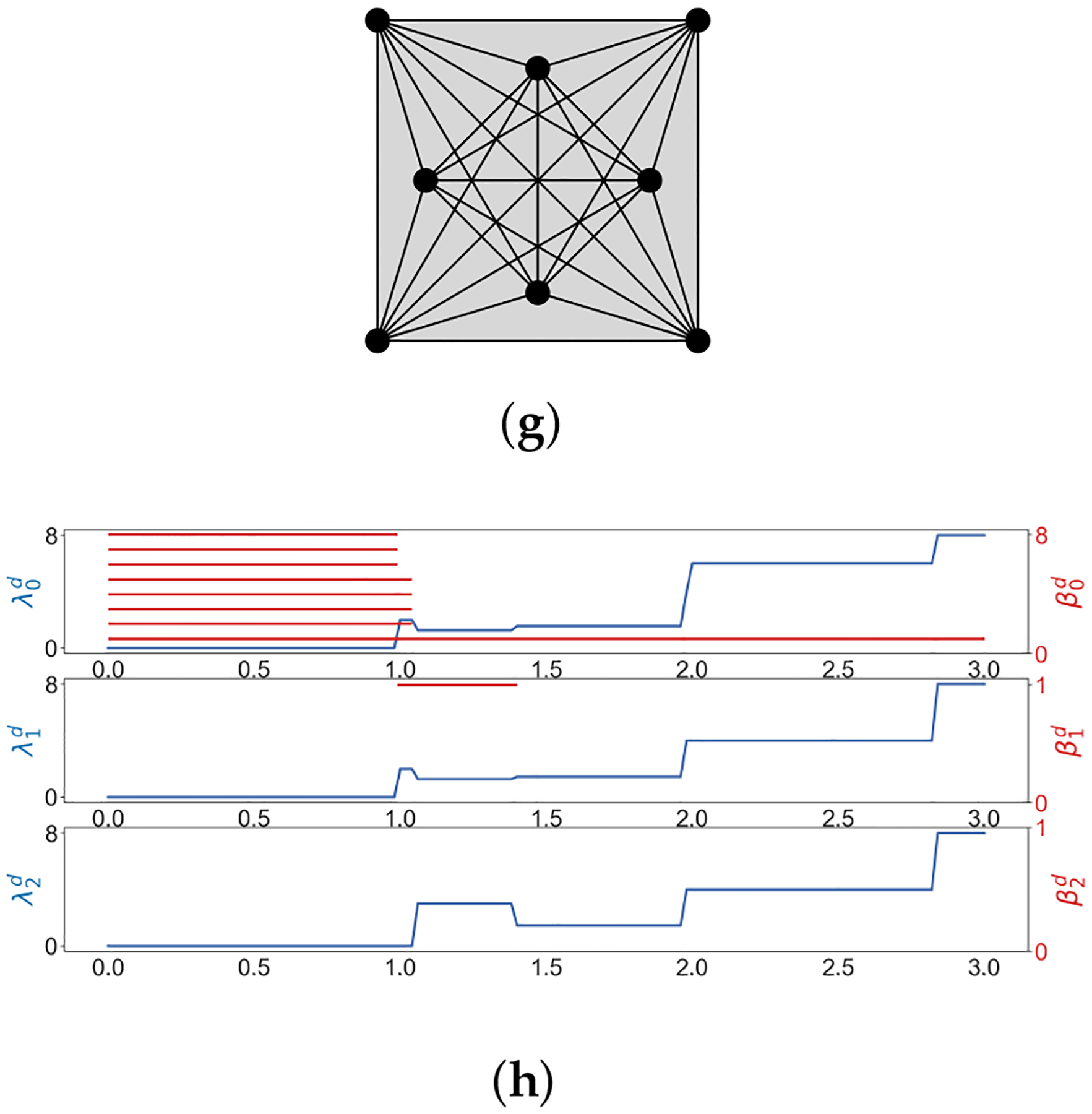
The Vietoris-Rips filtration of a point cloud, and some results of Laplacian calculation. (**a**) d=0; (**b**) d=0.99; (**c**) d=1.04; (**d**) d=1.40; (**e**) d=1.97; (**f**) d=2.00;(**g**) d=2.83; (**h**) The horizontal axis represents the diameter d (stepsize is 0.02). $\lambda_{q}^{d}$ (shown in blue lines) is the minimal nonzero eigenvalue of the q-th combinatorial Laplacian of $X_{d}$, and red bars represent homology classes that persist over d.

**Example 4.** *We illustrate the Vietoris-Rips filtration of a point cloud in* Figure 4. *Some results of Laplacian calculation are shown in* Figure 4h, *where* d *is the diameter (stepsize is 0.02)*, $\lambda_{q}^{d}$ *is the minimal nonzero eigenvalue of the* q-*th combinatorial Laplacian of* $X_{d}$, *and red bars represent homology classes that persist over* d. *The minimal nonzero eigenvalues change at different* d, *indicating the formation of new simplices*.

**Figure 5.**
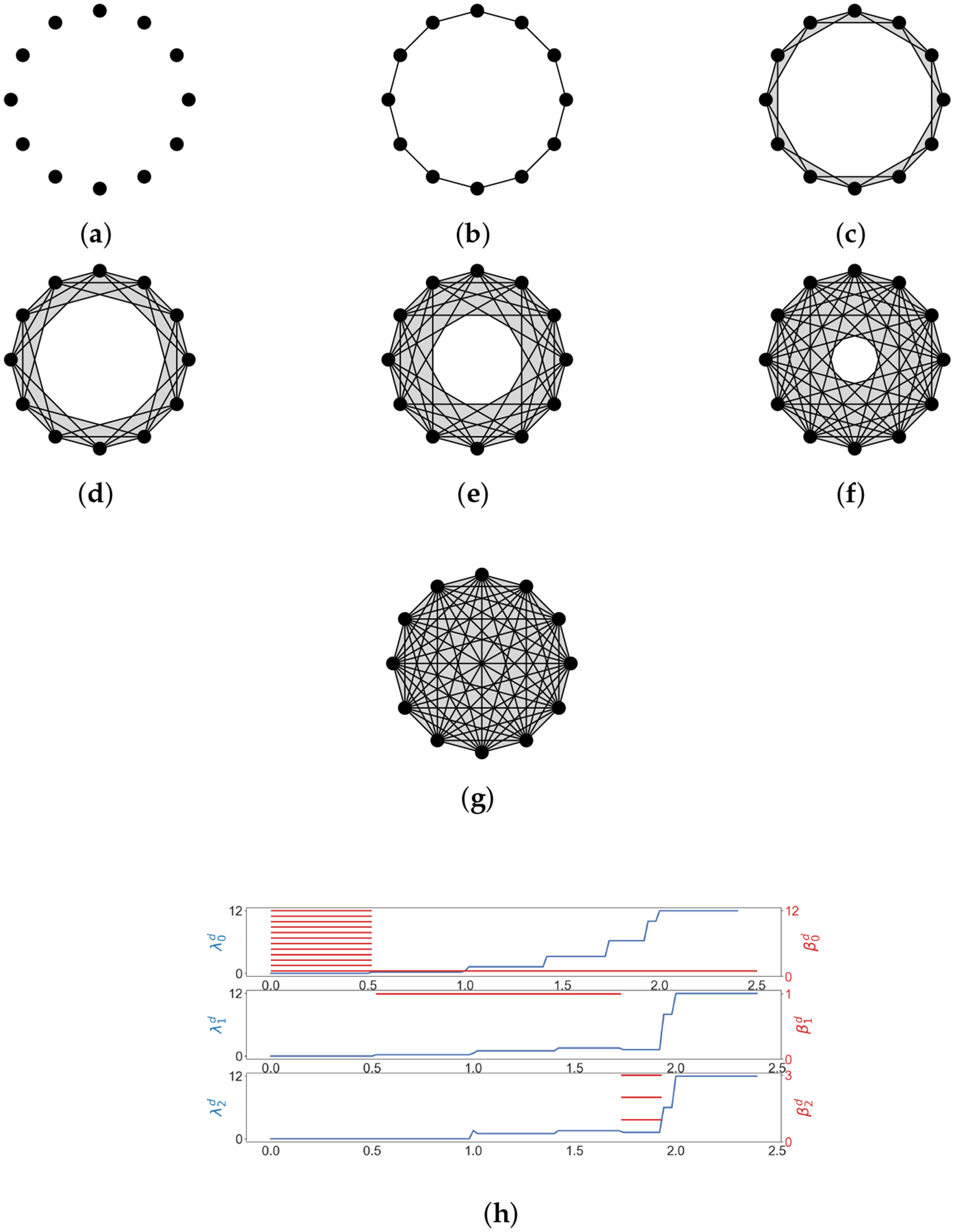
The Vietoris-Rips filtration of the vertices of a regular 12-gon, and some results of Laplacian calculation. (**a**) d=0; (**b**) d=0.52; (**c**) d=1.00; (**d**) d=1.42; (**e**) d=1.74; (**f**) d=1.94; (**g**) d=2.00; (**h**) The horizontal axis represents the diameter d (stepsize is 0.02). $\lambda_{q}^{d}$ (shown in blue lines) is the minimal nonzero eigenvalue of the q-th combinatorial Laplacian of $X_{d}$, and red bars represent homology classes that persist over d.

**Example 5.** *We consider the Vietoris-Rips filtration of the 12 vertices of the regular 12-gon* (Figure 5). *We see that* $\lambda_{2}^{d}$ *goes up before any homology class is born, which means that 2-simplices form but no 2-dimensional holes are born yet. This phenomenon can be observed in many results of* [63].

### 3.2. Matrix Representations of a (Persistent) Laplacian

Since $C_{q}$ has a canonical orthonormal basis, the matrix representation of $\partial_{q}^{*}$ is the transpose of the matrix representation of $\partial_{q}$. In the computation of persistent Laplacians, the difficult part is the calculation of the upper persistent Laplacian, because we need to determine $C_{q+1}^{X,Y}$, which may not have a canonical orthonormal basis. We can obtain a basis of $C_{q+1}^{X,Y}$ by performing a column reduction for $\partial_{q+1}^{Y}$ or by directly calculating the matrix representation of the upper persistent Laplacian by Schur complement [56]. We give two examples in the following.

**Figure 6.**
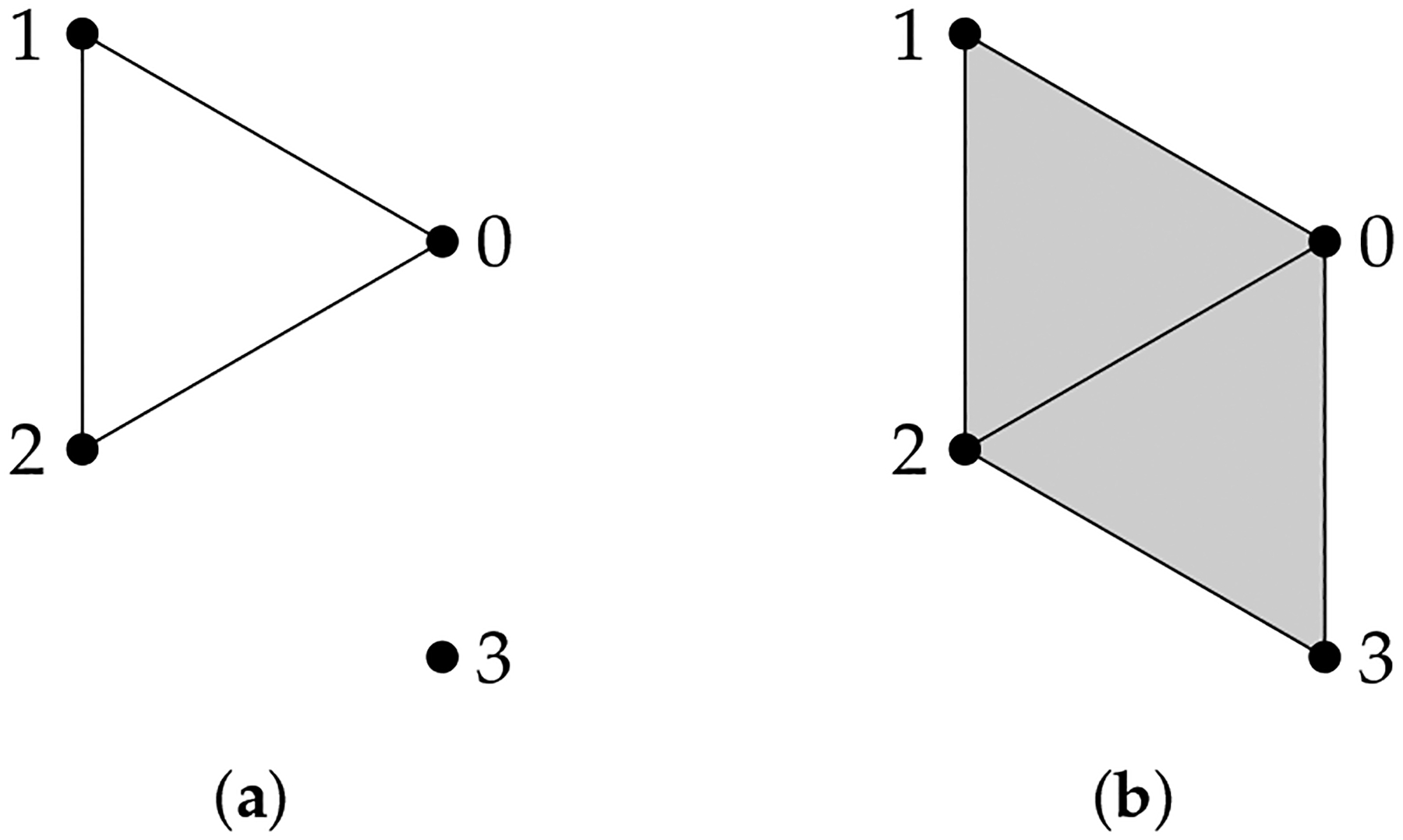
(**a**) X={0,1,2,3,01,12,02} and (**b**) Y={0,1,2,3,01,12,23,03,02,012,023}.

**Example 6.** *When* $C_{q+1}^{X,Y}$ is generated by some (q+1)-*simplices in* Y, *the calculation of $\partial_{q}^{X,Y}$* is relatively easy. For X *and* Y *shown in* Figure 6, *we compute* $\partial_{2}^{X,Y}$. *The matrix representation of $\partial_{2}^{Y}$* is

$$\begin{array}{cc} & \;012\;023\\ \begin{array}{c} 01\\ 12\\ 02\\ 23\\ 03 \end{array} & \left(\begin{array}{cc} 1 & 0\\ 1 & 0\\ -1 & 1\\ 0 & 1\\ 0 & -1 \end{array}\right), \end{array}$$

*so* $C_{2}^{X,Y}$ *is generated by* 012*; then, the matrix representation of* $\partial_{2}^{X,Y}$ *is*

$$\begin{array}{cc} & 012\\ \begin{array}{c} 01\\ 12\\ 02 \end{array} & \left(\begin{array}{c} 1\\ 1\\ -1 \end{array}\right) \end{array}.$$

**Figure 7.**
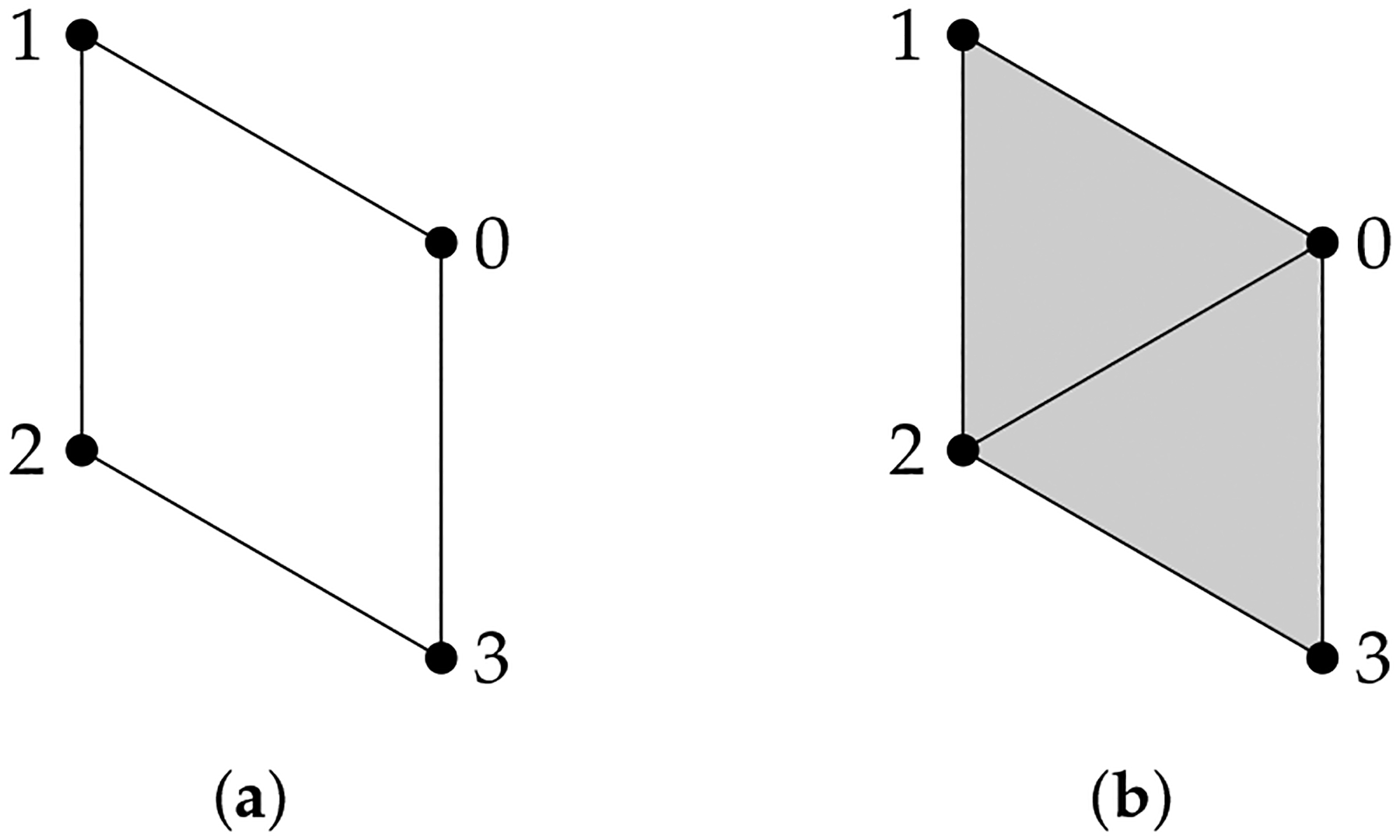
(**a**) X={0,1,2,3,01,12,23,03} and (**b**) Y={0,1,2,3,01,12,23,03,02,012,023}.

Example 7. We compute $\Delta_{1}^{X,Y}$ for X and Y shown in Figure 7.

*The matrix representation of $\partial_{2}^{Y}$ is*

$$\begin{array}{cc} & \begin{array}{cc} \;012 & 023 \end{array}\\ \begin{array}{c} 01\\ 12\\ 23\\ 03\\ 02 \end{array} & \left(\begin{array}{cc} 1 & 0\\ 1 & 0\\ 0 & 1\\ 0 & -1\\ -1 & 1 \end{array}\right) \end{array}.$$

*Our goal is to make the submatrix*

$$\begin{array}{cc} & \;012\;023\\ 02 & \left(\begin{array}{cc} -1 & 1 \end{array}\right) \end{array}$$

in column echelon form. We apply one column reduction and obtain

$$\begin{array}{cc} & \;012\;023+012\\ \begin{array}{c} 01\\ 12\\ 23\\ 03\\ 02 \end{array} & \left(\begin{array}{cc} 1 & \;1\\ 1 & \;1\\ 0 & \;1\\ 0 & \;-1\\ -1 & \;0 \end{array}\right). \end{array}$$

*Therefore*, $C_{2}^{X,Y}=\text{span}(023+012)$ *and one matrix representation of* $\partial_{2}^{X,Y}$ *is*

$$\begin{array}{cc} & 023+012\\ \begin{array}{c} 01\\ 12\\ 23\\ 03 \end{array} & \left(\begin{array}{c} 1\\ 1\\ 1\\ -1 \end{array}\right) \end{array}.$$

For any two spaces V *and* W *and* f:V→W, *if we choose arbitrary bases of* V and W *and take a matrix representation* $M_{f}$ *of* f, *then the matrix representation* $M_{f^{*}}$ *of* $f^{*}$ *is* $P^{-1}M_{f}^{T}Q$, *where* P *and* Q are inner product matrices of V *and* W, *respectively. If we use* {023+012} *as the basis of* $C_{2}^{X,Y}$, *then the inner product matrix of $C_{2}^{X,Y}$ is* 2 *(the norm of* 023+012). *The corresponding matrix representation of* ${\left(\partial_{2}^{X,Y}\right)}^{*}$ *is*

$$\frac{1}{2}\left(\begin{array}{llll} 1 & 1 & 1 & -1 \end{array}\right)$$

*and the matrix representation of the upper persistent Laplacian is*

$$\begin{array}{cc} & \;01\;12\;23\;03\\ \begin{array}{cc} \frac{1}{2} & \begin{array}{c} 01\\ 12\\ 23\\ 03 \end{array} \end{array} & \left(\begin{array}{cccc} 1 & 1 & 1 & -1\\ 1 & 1 & 1 & -1\\ 1 & 1 & 1 & -1\\ -1 & -1 & -1 & 1 \end{array}\right) \end{array}.$$

*Another way to compute the upper persistent Laplacian is as follows. We first compute the upper Laplacian* $\Delta_{1}(Y)$

$$\begin{array}{cc} & \begin{array}{ccccc} 01 & 12 & 23 & 03 & 02 \end{array}\\ \begin{array}{c} 01\\ 12\\ 23\\ 03\\ 02 \end{array} & \left(\begin{array}{ccccc} 1 & 1 & 0 & 0 & -1\\ 1 & 1 & 0 & 0 & -1\\ 0 & 0 & 1 & -1 & 1\\ 0 & 0 & -1 & 1 & -1\\ -1 & -1 & 1 & -1 & 2 \end{array}\right) \end{array},$$

*then treat this matrix as a block matrix*

$$\begin{array}{cc} & \;C_{1}(X)\;02\\ \begin{array}{c} C_{1}(X)\\ 02 \end{array} & \left(\begin{array}{cc} \;A & \;B\\ \;C & \;D \end{array}\right), \end{array}$$

*and compute the Schur complement* $A-BD^{-1}C$.

### 3.3. Eigenvectors of a Laplacian

There are some results concerning the relationship between the spectra of Laplacians and the shape of a simplicial complex [26,79]. How do we interpret the eigenvectors of a Laplacian? For an eigenvector of a q-th combinatorial Laplacian, we can look at the shape of q-simplices where the eigenvector has support (signs are arbitrary because they are affected by the fixed ordering of vertices). Empirical observations [80–82] suggest that (a) harmonic eigenvectors (eigenvectors of zero eigenvalues) have support near q-dimensional “holes” (or vertices in a connected component when q=0) or (b) nonharmonic eigenvectors (eigenvectors of nonzero eigenvalues) have support near “clusters” of q-simplices. In terms of persistent Laplacians, very little is known about the topological interpretation of eigenvalues and eigenvectors.

## 4. Generalizations of (Persistent) Laplacians

From a theoretical point of view, it is natural to ask whether a persistent Laplacian can be defined in other settings such that the persistent Hodge theorem still holds. From a practical point of view, generalizations of persistent Laplacians are motivated by the need to integrate non-geometrical data that are important for specific problems. In the next few sections, we will introduce structures such as cellular (co)sheaves, digraphs, and hyper(di)graphs, and then review some recent advances in their homology and Laplacians. We will also discuss Dirac operators and N-chain complexes at the end of this section.

### 4.1. Differential Graded Inner Product Spaces

It has been noted earlier that persistent Laplacians can be defined analogously for differential graded inner product spaces and the persistent Hodge theorem can be proved. A differential graded inner product space (V,d) is just a chain complex

$$\cdots \overset{d_{q+2}\;}{\to }V_{q+1}\overset{d_{q+1}\;}{\to }V_{q}\overset{d_{q}\;}{\to }V_{q-1}\overset{d_{q-1}\;}{\to }\cdots$$

whose chain groups are inner product spaces. When we say $\left(V,d^{V}\right)$ is a subspace of $\left(W,d^{W}\right)$, we mean that the inner space structure and boundary operator $d^{V}$ of $\left(V,d^{V}\right)$ are inherited from $\left(W,d^{W}\right)$. For a pair of differential graded inner product spaces $\left(V,d^{V}\right)\subset \;\left(W,d^{W}\right)$, the q-th persistent homology group is defined analogously by

$$\iota^{•}\left(H_{q}(V)\right)≅\frac{\text{ker}\;d_{q}^{V}}{\text{ker}\;d_{q}^{V}\cap \text{im}\;d_{q+1}^{W}}.$$

Observe that $\text{ker}\;d_{q}^{V}\cap \text{im}\;d_{q+1}^{W}=V_{q}\cap \text{im}\;d_{q+1}^{W}$. The preimage of $V_{q}\cap \text{im}\;d_{q+1}^{W}$ under $d_{q+1}^{W}$ is just ${\left(d_{q+1}^{W}\right)}^{-1}\left(V_{q}\right)=\left\{w\in W_{q+1}|d_{q+1}^{W}w\in V_{q}\right\}$. Hence, $\text{ker}\;d_{q}^{V}\cap \text{im}\;d_{q+1}^{W}$ is the image of ${\left.\pi d_{q+1}^{W}\right|}_{{\left(d_{q+1}^{W}\right)}^{-1}\left(V_{q}\right)}:{\left(d_{q+1}^{W}\right)}^{-1}\left(V_{q}\right)\to V_{q}$, where $\pi =\iota^{†}$ is the projection map from W to V. We denote ${\left.\pi d_{q+1}^{W}\right|}_{{\left(d_{q+1}^{W}\right)}^{-1}\left(V_{q}\right)}$ by $d_{q+1}^{V,W}$, and ${\left(d_{q+1}^{W}\right)}^{-1}\left(V_{q}\right)$ by $\Theta_{q+1}^{V,W}$. These maps are shown in the following diagram:


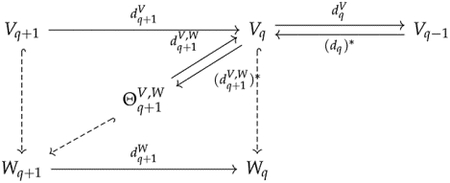




where hooked dashed arrows represent inclusion maps. We define the q-th persistent Laplacian $\Delta_{q}^{V,W}:V_{q}\to V_{q}$ by

$${\left(d_{q}^{V}\right)}^{*}d_{q}^{V}+d_{q+1}^{V,W}{\left(d_{q+1}^{V,W}\right)}^{*}.$$

Since $d_{q}^{V}d_{q+1}^{V,W}=0$, we can prove the persistent Hodge theorem

$$\text{ker}\;\Delta_{q}^{V,W}≅\frac{\text{ker}\;d_{q}^{V}}{\text{ker}\;d_{q}^{V}\cap \text{im}\;d_{q+1}^{W}}$$

in a similar manner. Many generalizations of persistent Laplacians implicitly use this formulation. Liu et al. [55] first defined persistent Laplacians in the setting of differential graded inner product spaces and showed how to construct a persistent Laplacian for an inner product preserving chain map.

### 4.2. Persistent Laplacians for Simplicial Maps

The classical filtration of simplicial complexes only represents one type of shape evolution. We also need tools to study more general shape evolution, such as the sparsification of a simplicial complex. This requires us to consider general simplicial maps rather than inclusion maps. Gülen et al. [54] developed a theory of persistent Laplacians for a simplicial map. Suppose f:X→Y is a simplicial map


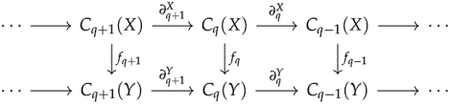




where $f_{q}:C_{q}(X)\to C_{q}(Y)$ is induced by f. Different from the original q-th persistent Laplacian for an inclusion map, we need to define two subspaces

$$C_{q+1}(Y)⊃C_{q+1}^{Y\leftarrow X}=\left\{c\in C_{q+1}(Y)|\partial_{q+1}^{Y}(c)\in f_{q}\left(\text{ker}\;\partial_{q}^{X}\right)\right\}$$

and

$$C_{q-1}(X)⊃C_{q-1}^{X\to Y}=\left\{c\in C_{q-1}^{X}|{\left(\partial_{q}^{X}\right)}^{*}(c)\in {\left(\text{ker}f_{q}\right)}^{⊥}\right\}$$

and then apply the restrictions of $\partial_{q+1}^{Y}$ and ${\left(\partial_{q}^{X}\right)}^{*}$ to them to construct the q-th persistent Laplacian for f. The q-th persistent Laplacian for a simplicial map has a more symmetric expression, and the proof of persistent Hodge theorem is more complicated.

### 4.3. Weighted Simplicial Complexes

A simplicial complex whose simplices have weights is generally called a weighted simplicial complex. The weights can be geometrical, such as angles between simplices, volumes of simplices, or non-geometrical such as numbers of scientific papers coauthored by groups of people. Many theories and models involving weighted simplicial complexes exist (e.g., [83–88]). Here, we focus on the theory of weighted simplicial complexes proposed by Dawson [89] and later developed in [90–96]. A weighted simplicial complex is a simplicial complex where each simplex σ has a weight w(σ) valued in a commutative ring R, such that if σ⩽τ, then w(τ) is divisible by w(σ). The weighted chain complex of a weighted simplicial complex X is defined as follows. Let $C_{q}(X,w)$ be the set of formal sums of q-simplices with coefficients in R (if w(σ) is zero, then we do not include σ in any formal sum). For $\sigma =\left[v_{a_{0}},\ldots ,v_{a_{q}}\right]$, we denote the face $\left[v_{a_{0}},\ldots ,{\overset{ˆ}{v}}_{a_{i}},\ldots ,v_{a_{q}}\right]$ by $d_{i}\sigma$. The weighted boundary operator ∂ is given by

$$\partial (\sigma )=\sum_{i=0}^{q}\frac{w(\sigma )}{w\left(d_{i}\sigma \right)}(-1{)}^{i}d_{i}\sigma .$$

As w(σ) is divisible by $w\left(d_{i}\sigma \right)$, the weighted boundary operator is well defined. We still have $\partial^{2}=0$, because for 0≤i<j≤q,

$$\frac{w(\sigma )}{w\left(d_{i}\sigma \right)}\frac{w\left(d_{i}\sigma \right)}{w\left(d_{j-1}d_{i}\sigma \right)}=\frac{w(\sigma )}{w\left(d_{j}\sigma \right)}\frac{w\left(d_{j}\sigma \right)}{w\left(d_{i}d_{j}\sigma \right)}=\frac{w(\sigma )}{w\left(d_{i}d_{j}\sigma \right)}.$$

Therefore, weighted homology groups can be defined analogously. Wu et al. [95] pointed out that in the proof of $\partial^{2}=0$, what really matters is the quotient of weights. If we write w(τ)/w(σ) as ϕ(τ,σ), then the equality

$$\frac{w(\sigma )}{w\left(d_{i}\sigma \right)}\frac{w\left(d_{i}\sigma \right)}{w\left(d_{j-1}d_{i}\sigma \right)}=\frac{w(\sigma )}{w\left(d_{j}\sigma \right)}\frac{w\left(d_{j}\sigma \right)}{w\left(d_{i}d_{j}\sigma \right)}$$

becomes

$$\phi \left(d_{i}\sigma ,d_{j-1}d_{i}\sigma \right)\phi \left(\sigma ,d_{i}\sigma \right)=\phi \left(d_{j}\sigma ,d_{i}d_{j}\sigma \right)\phi \left(\sigma ,d_{j}\sigma \right),$$

which means that any ϕ:X×X→R satisfying this equality induces a (ϕ-weighted) boundary operator

$$\partial_{q}(\sigma )=\sum_{i=0}^{q}(-1{)}^{i}\phi \left(\sigma ,d_{i}\sigma \right)d_{i}\sigma .$$

A simplicial complex paired with a generalized weight function ϕ is called a ϕ-weighted simplicial complex.

**Figure 8.**
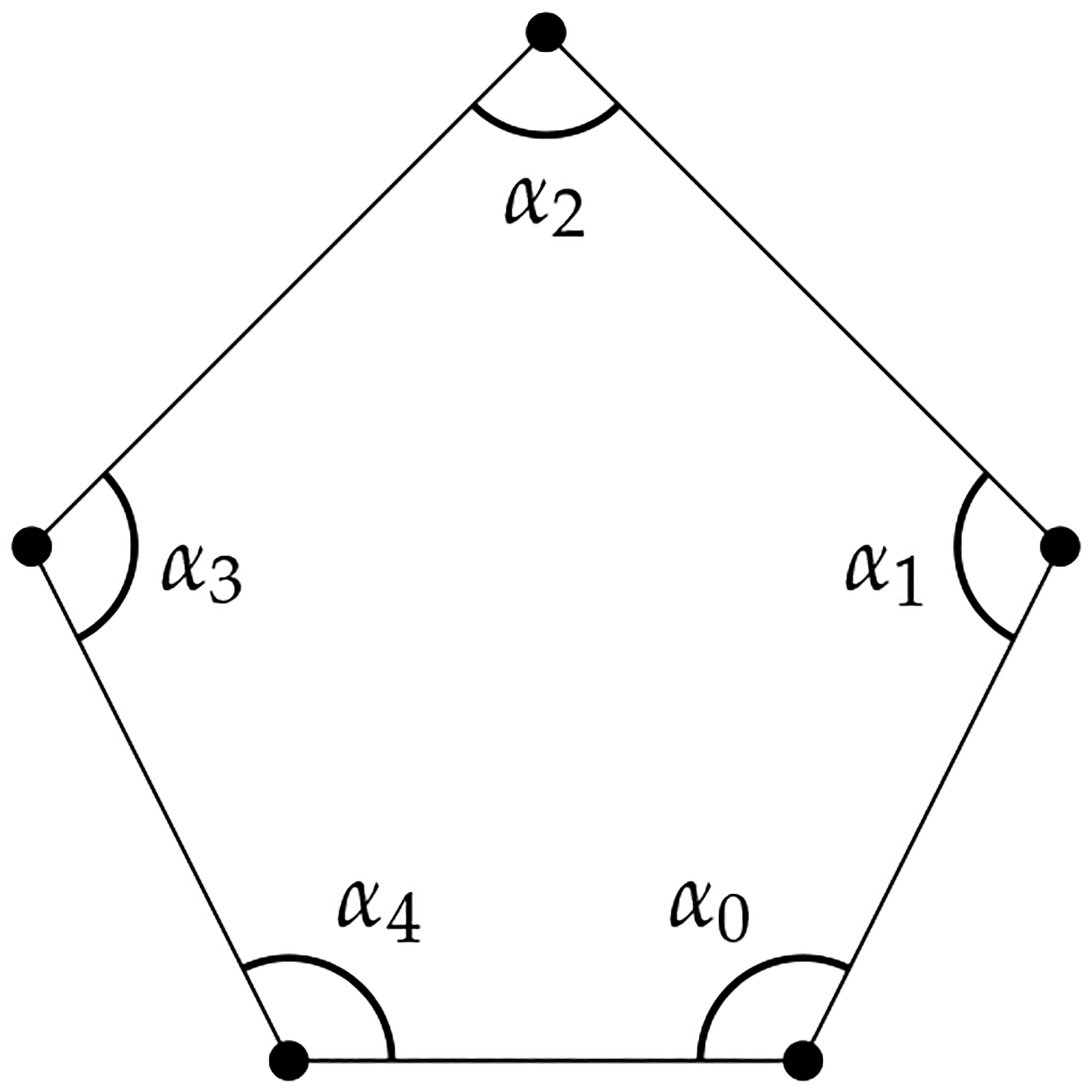
A weighted polygon.

**Example 8** ([95]). *A weighted polygon is a polygon with* $\phi \left(\left\{v_{i},v_{j}\right\},v_{i}\right)=\alpha_{i}\in Z$ (Figure 8). *The matrix representation of* $\partial_{1}$ *is*

$$\begin{array}{cc} & \begin{array}{ccccc} v_{0}v_{1} & v_{0}v_{4} & v_{1}v_{2} & v_{2}v_{3} & v_{3}v_{4} \end{array}\\ \begin{array}{c} v_{0}\\ v_{1}\\ v_{2}\\ v_{3}\\ v_{4} \end{array} & \left(\begin{array}{ccccc} -\alpha_{0} & -\alpha_{0} & 0 & 0 & 0\\ \alpha_{1} & 0 & -\alpha_{1} & 0 & 0\\ 0 & 0 & \alpha_{2} & -\alpha_{2} & 0\\ 0 & 0 & 0 & \alpha_{3} & -\alpha_{3}\\ 0 & \alpha_{4} & 0 & 0 & \alpha_{4} \end{array}\right) \end{array}$$

*and the resulting weighted $H_{0}$ is dependent on $\alpha_{i}$*. *The weighted homology of weighted polygons might be useful for analyzing ring structures in biomolecules*.

We have emphasized that a point cloud can be studied by building a filtration of simplicial complexes. If we want to distinguish some points from other points, we can assign weights and building a filtration of weighted simplicial complexes [94]. We may also consider weighted versions of (persistent) Laplacians [95].

**Example 9.** *Suppose each point* v *in a point cloud has weight* w(v). *We can associate any simplex* $\left\{v_{a_{0}},\ldots ,v_{a_{q}}\right\}$ *as the product weight* [94]

$$\prod_{i=0}^{q}w\left(v_{a_{i}}\right).$$

*Since the weighted boundary map can be given by*

$$\partial (\sigma )=\sum_{i=0}^{q}w\left(v_{a_{i}}\right)(-1{)}^{i}d_{i}\sigma .$$

*We can just define the* q-*th chain group as the space generated by* q-*simplices without worrying about zero weights*.

**Figure 9.**
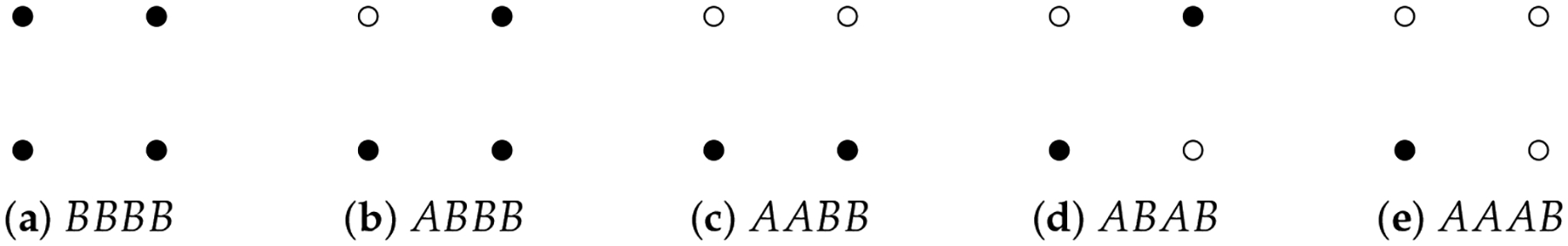
Five configurations of A and B.

**Figure 10.**
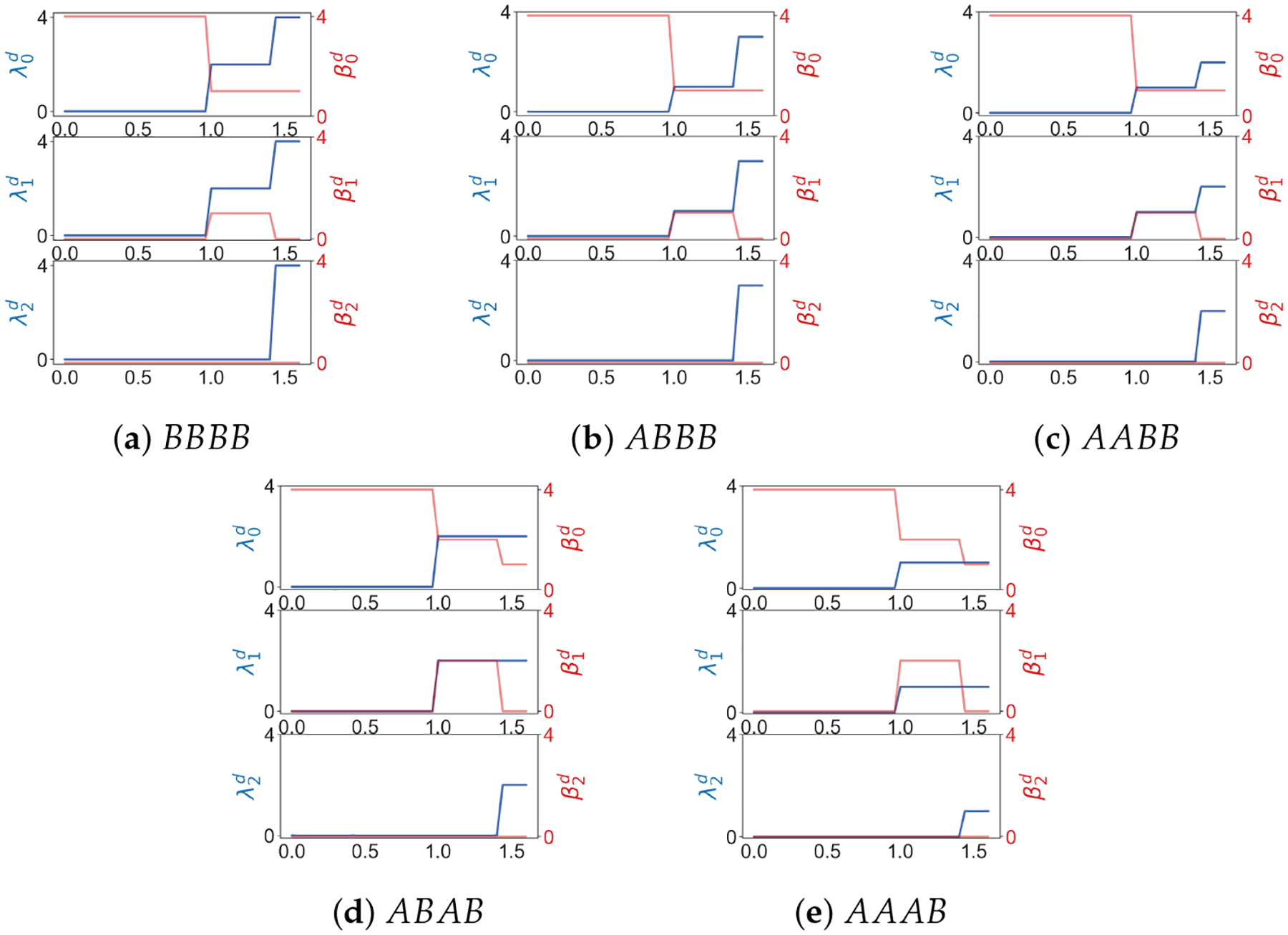
$\lambda_{q}^{d}$ is the minimal nonzero eigenvalue of the q-th weighted combinatorial Laplacian for $X_{d}$ in a Rips filtration. $\beta_{q}^{d}$ is the q-th Betti number of $X_{d}$.

**Example 10.** *Suppose a point cloud contains two types of points* A *and* B. *We can assign weights* {0, 1} *to* {A,B}, *and compute weighted homology and Laplacians using product weighting. At least when a point cloud is simple, weighted combinatorial Laplacians can be used to differentiate among different patterns of distribution of* A *and* B. *For a point cloud of four points* {(0, 0), (1, 0), (1, 1), (0, 1)} *there are five configurations (shown in* Figure 9) *that include at least one point whose weight is 1*. *Results of weighted Laplacians are shown in* Figure 10.

### 4.4. Cellular (Co)sheaves

In a ϕ-weighted simplicial complex, we can imagine that a copy of R resides on each simplex and ϕ(τ,σ) is a scalar multiplication from the copy on τ to the copy on σ [97]. If we associate each simplex with a vector space and designate a linear morphism for every face relation, we will obtain cellular (co)sheaf. The theory of cellular (co)sheaves was first introduced in [98] and later gained attention for its application potential (e.g., [99–103]). In recent years, the study of sheaf neural networks has become a trending topic [104–107]. Like a weighted simplicial complex, a cellular (co)sheaf is a candidate for modeling complex objects such as molecules.

A cellular cosheaf ℱ is a simplicial complex X with additional data (for ease of exposition, we have simplified the definition of a cellular (co)sheaf). Each simplex σ is assigned a vector space ℱ(σ) (or denoted by ${ℱ}_{\sigma }$), called the stalk over σ, and for any face relation σ⩽τ, there is an extension map ℱ(σ⩽τ):ℱ(τ)→ℱ(σ) (or denoted by ${ℱ}_{\sigma ⩽\tau }$). The q-th chain group of a cellular cosheaf is the direct sum of stalks over q-simplices and the boundary map ∂ is given by

$${\left.\partial_{q}\right|}_{ℱ(\sigma )}=\sum_{i}(-1{)}^{i}ℱ\left(d_{i}\sigma ⩽\sigma \right).$$

The square of this boundary map is 0, if for any face relation ρ⩽σ⩽τ we have

ℱ(ρ⩽τ)=ℱ(ρ⩽σ)∘ℱ(σ⩽τ).

A dual concept is a cellular sheaf. For a cellular sheaf ℱ,ℱ(σ⩽τ) is a map from ℱ(σ) to ℱ(τ) (called a restriction map). An analogous sheaf cochain complex can be defined. If stalks are inner product spaces, one can equip inner product structures for (co)chain groups. Applying the construction of combinatorial Laplacians to a cosheaf chain complex or a sheaf cochain complex, we obtain (co)sheaf Laplacians [97]. It is noted that many sheaves over a digraph only have trivial 0-dimensional cohomology groups [108], but we can still extract some information from sheaf Laplacians.

**Example 11.** *Suppose there is a sheaf* ℱ *over the simplicial complex* {0, 1, 2, 01, 02, 12}, *then the sheaf coboundary map $\delta^{0}$ is represented by the block matrix*

$$\begin{array}{l} \begin{array}{ccc} \;{ℱ}_{0} & \;{ℱ}_{1} & \;{ℱ}_{2} \end{array}\\ \begin{array}{cc} \begin{array}{c} {ℱ}_{01}\\ {ℱ}_{02}\\ {ℱ}_{12} \end{array} & \left(\begin{array}{ccc} -{ℱ}_{0⩽01} & {ℱ}_{1⩽01} & 0\\ -{ℱ}_{0⩽02} & 0 & {ℱ}_{2⩽02}\\ 0 & -{ℱ}_{1⩽12} & {ℱ}_{2⩽12} \end{array}\right). \end{array} \end{array}$$

*Suppose all stalks are inner product spaces and they are orthogonal to each other, then the* 0-*th sheaf Laplacian* $\delta^{0^{*}}\delta^{0}$ *is represented by the block matrix*

$$\begin{array}{l} \begin{array}{ccc} \;{ℱ}_{0} & \;{ℱ}_{1} & \;{ℱ}_{2} \end{array}\\ \begin{array}{cc} \begin{array}{c} {ℱ}_{0}\\ {ℱ}_{1}\\ {ℱ}_{2} \end{array} & \left(\begin{array}{ccc} {ℱ}_{0⩽01}^{*}{ℱ}_{0⩽01}+{ℱ}_{0⩽02}^{*}{ℱ}_{0⩽02} & -{ℱ}_{0⩽01}^{*}{ℱ}_{1⩽01} & -{ℱ}_{0⩽02}^{*}{ℱ}_{2⩽02}\\ -{ℱ}_{1⩽01}^{*}{ℱ}_{0⩽01} & {ℱ}_{1⩽01}^{*}{ℱ}_{1⩽01}+{ℱ}_{1⩽12}^{*}{ℱ}_{1⩽12} & -{ℱ}_{1⩽12}^{*}{ℱ}_{2⩽12}\\ -{ℱ}_{2⩽02}^{*}{ℱ}_{0⩽02} & -{ℱ}_{2⩽12}^{*}{ℱ}_{1⩽12} & {ℱ}_{2⩽02}^{*}{ℱ}_{2⩽02}+{ℱ}_{2⩽12}^{*}{ℱ}_{2⩽12} \end{array}\right). \end{array} \end{array}$$

Persistent (co)sheaf (co)homology is known by experts [109,110], and a systematic treatment can be found in [111]. One type of filtration of sheaves is as follows. A sheaf ℱ on X is a “subsheaf” of 𝒢 over Y if X⊂Y and the stalks and restriction maps of ℱ are the same as those of 𝒢. To define the q-th persistent sheaf Laplacian [59] for ℱ⊂𝒢, we can first endow chain groups with inner product structures and dualize everything to make ℱ and 𝒢 cosheaves, such that there is an inclusion chain map between their cosheaf chain complexes. Then, we can define the q-th persistent sheaf Laplacian as the q-th persistent Laplacian of the cosheaf chain complexes.

### 4.5. Path Homology, Flag Homology, and Digraphs

The motivation behind path homology is to construct a homology theory of digraphs such that directional information of edges is encoded and higher-dimensional homology groups are non-trivial. Path homology (there are other (co)homology theories of digraphs [112–115]) was proposed by Grigor’yan, Lin, Muranov, and Yau [116] and developed in various papers [117–121]. A summary of recent advances in path homology of digraphs can be found in [122]. Recall that a digraph (without self-loops) is a pair G=(V,E) where E is a set of ordered pairs of vertices. An allowed q-path is an ordered finite sequence of vertices $\left\{x_{0},\ldots ,x_{q}\right\}$ such that $\left(x_{i},x_{i+1}\right)\in E$ for all 0≤i≤q-1. If we take the space generated by allowed p-paths (denoted by ${𝒜}_{q}$) as the q-th chain group, and define the boundary map $\partial_{q}$ by

$$\partial_{q}\left\{x_{0},\ldots ,x_{q}\right\}=\sum_{i=0}^{q}(-1{)}^{i}\left\{x_{0},\ldots ,{\overset{ˆ}{x}}_{i},\ldots ,x_{q}\right\}$$

then formally we can show that $\partial^{2}=0$. However, $\partial_{q}\left\{x_{0},\ldots ,x_{q}\right\}$ may include paths that are not allowed. To solve this problem, we first need to introduce some general concepts.

**Definition 1.** Suppose X *is a finite set. An elementary* p-path is a sequence $\left[x_{0},\ldots ,x_{p}\right]$ *of* p+1 *elements of* X. *The space generated by all elementary* p-*paths with coefficient in* R *is denoted by* $\Lambda_{p}(X)$. *The* q-*th non-regular boundary map is given by*

$$\partial_{q}^{nr}\left[x_{0},\ldots ,x_{q}\right]=\sum_{i=0}^{p}\left[x_{0},\ldots ,{\overset{ˆ}{x}}_{i},\ldots ,x_{q}\right].$$

We can prove that this is a chain complex. Among all paths, a path that lingers at a vertex (for some $i,x_{i}=x_{i+1}$) is considered a degenerate path, since we are not interested in self-loops.

**Definition 2.** *A path* $\left[x_{0},\ldots ,x_{q}\right]$ *over* X *where* $x_{i}\neq x_{i+1}$ *for each* i *is called regular*. *The space generated by all regular* q-*paths is denoted by* ${ℛ}_{q}$.

We define a new boundary operator $\partial_{q}$ *between regular paths. When computing* $\partial_{q}\left(\left[x_{0},\ldots ,x_{q}\right]\right)$, *we first compute* $\partial_{q}^{\text{nr}}\left(\left[x_{0},\ldots ,x_{q}\right]\right)$ and treat all irregular paths arising from it as zeros. We can still verify that $\partial^{2}=0$ [123].

Now, given a digraph G=(V,E), every ${𝒜}_{q}$ is a subspace of ${ℛ}_{q}$.


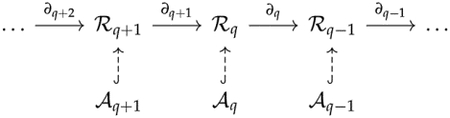




One way to make $\partial_{q}:{𝒜}_{q}\to {𝒜}_{q-1}$ well-defined is to restrict $\partial_{q}$ to the subspace ${𝒜}_{q}\cap \partial_{q}^{-1}{𝒜}_{q-1}$. We have to verify that $\partial_{q}\left({𝒜}_{q}\cap \partial_{q}^{-1}{𝒜}_{q-1}\right)\subset {𝒜}_{q-1}\cap \partial_{q-1}^{-1}{𝒜}_{q-2}.\;\partial_{q}\left({𝒜}_{q}\cap \right.\;\left.\partial_{q}^{-1}{𝒜}_{q-1}\right)\subset {𝒜}_{q-1}\cap$ is true by definition and $\partial_{q}\left({𝒜}_{q}\cap \partial_{q}^{-1}{𝒜}_{q-1}\right)\subset \partial_{q-1}^{-1}{𝒜}_{q-2}$ is true since $\partial^{2}=0$. Therefore, we have the chain complex

$$\ldots ⟶{𝒜}_{q+1}\cap \partial_{q+1}^{-1}{𝒜}_{q}\overset{\partial_{q+1}\;}{\to }{𝒜}_{q}\cap \partial_{q}^{-1}{𝒜}_{q-1}\overset{\partial_{q}\;}{\to }{𝒜}_{q}\cap \partial_{q}^{-1}{𝒜}_{q-1}⟶\ldots$$

and the definition of a path homology group is straightforward. The q-th chain group ${𝒜}_{q}\cap \partial_{q}^{-1}{𝒜}_{q-1}$ is called the space of ∂-invariant q-paths on G, denoted by $\Omega_{q}$ (if a digraph is not simple, there will be two choices of $\partial_{q}$ [116] that might be suitable for different problems [124]). Regarding the geometrical interpretation of path homology, we only know that non-reduced $H_{0}$ is the number of connected components of the underlying undirected graph. Chowdhury et al. [125] obtained some characterizations of path homologies of certain families of small digraphs. Since edge direction information is encoded in path homology, path homology can be used to distinguish network motifs [126] and isomers in molecular and materials sciences [127]. We can also quantify the importance of a node in a network by observing changes in path homology when the node is removed [127].

**Figure 11.**
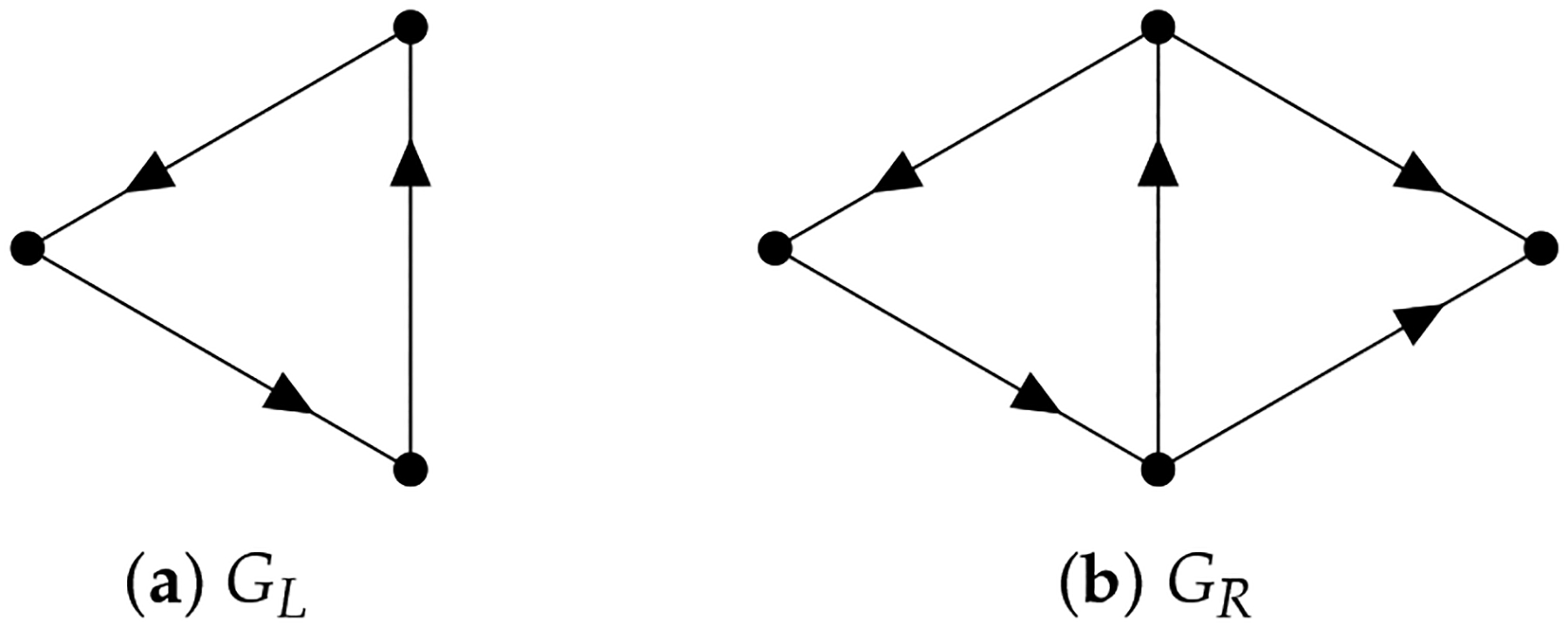
Two digraphs that have the same path homology.

Since $\Omega_{q}$ inherits the inner product structure from ${𝒜}_{q}$, the so-called path Laplacians (another type of path Laplacians was proposed by Estrada [128] and applied in molecular biology [129]) can be defined. We can use path Laplacians [58,122,130] to distinguish among digraphs that have the same path homology. For example, according to ([116], Theorem 5.4), the following two digraphs $G_{L}$ and $G_{R}$ (see Figure 11) have the same path homology. However, the spectrum of the 0-th path Laplacian of $G_{L}$ is {0, 3, 3} and that of $G_{R}$ is {0, 2, 4, 4}.

Persistent path homology was proposed by Chowdhury and Mémoli [126] to study a digraph where each edge e has a weight w(e). A filtration of digraphs $\left\{G_{d}\right\}$ is constructed so that $e\in G_{d}$ if and only if w(e)≤d. Wang and Wei [58] introduced persistent path Laplacians and demonstrated that persistent path Laplacians can be applied to the study of molecules, since much information about molecules can be encoded in digraphs.

Flag complexes, also known as clique complexes, are another way to construct homology for digraphs, and they arise naturally in many situations [113]. Jones and Wei [57] introduced persistent directed flag Laplacians as a distinct way of analyzing flag complexes and applied them to analyze protein-ligand binding data.

**Figure 12.**
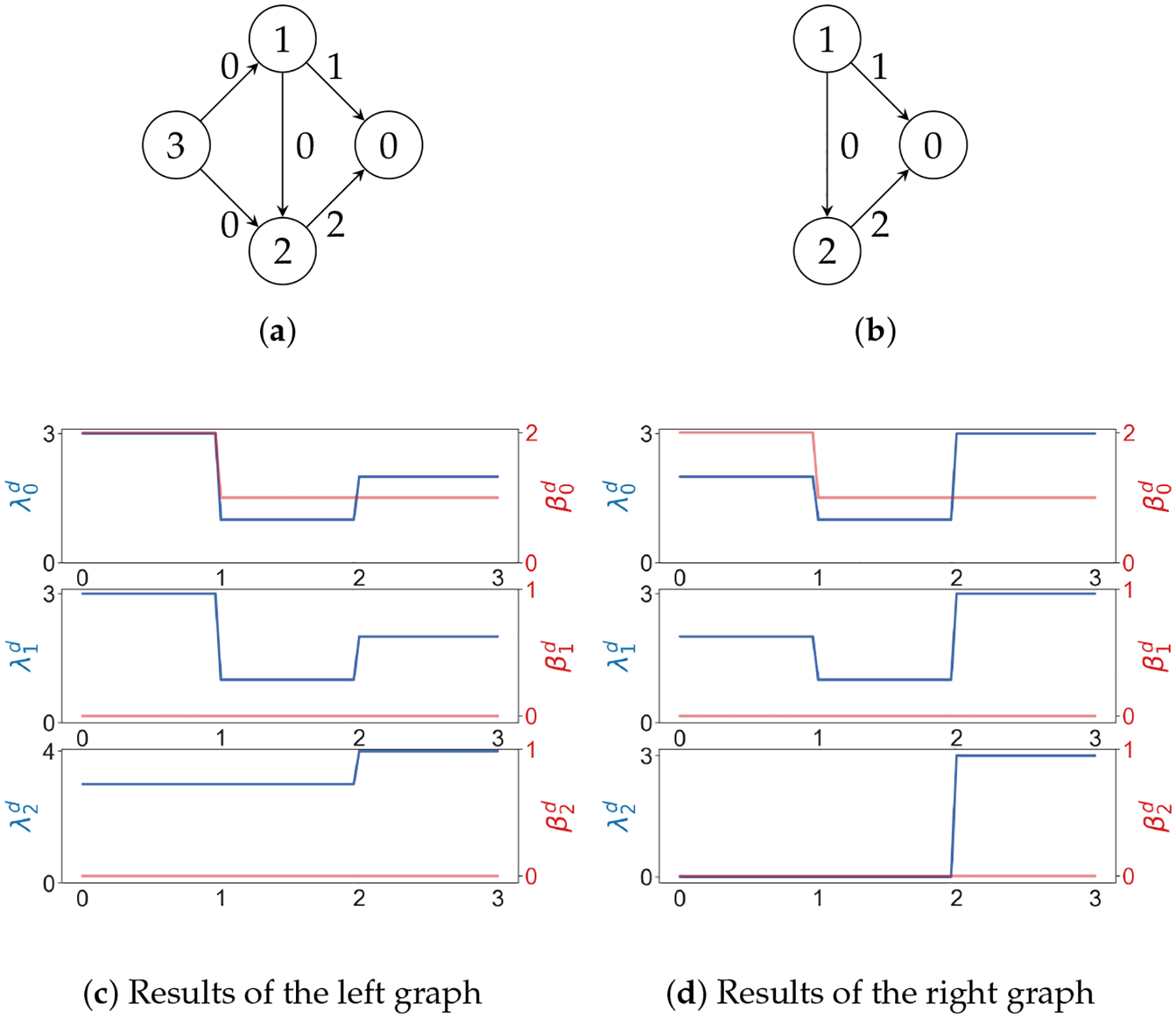
In (**a**,**b**), numbers on edges are weights. In (**c**,**d**), the x axis represents the weight. As usual, λ and β represent the minimal nonzero eigenvalues and Betti numbers.

**Example 12.** *For a weighted digraph, we can build a filtration* $\left\{G_{d}\right\}$ *such that* $e\in G_{d}$ *iff* w(e)≤d. *Two weighted graphs whose path Betti numbers are the same for every* $G_{d}$ *may have different path Laplacians* (Figure 12).

### 4.6. Hypergraphs and Hyperdigraphs

**Figure 13.**
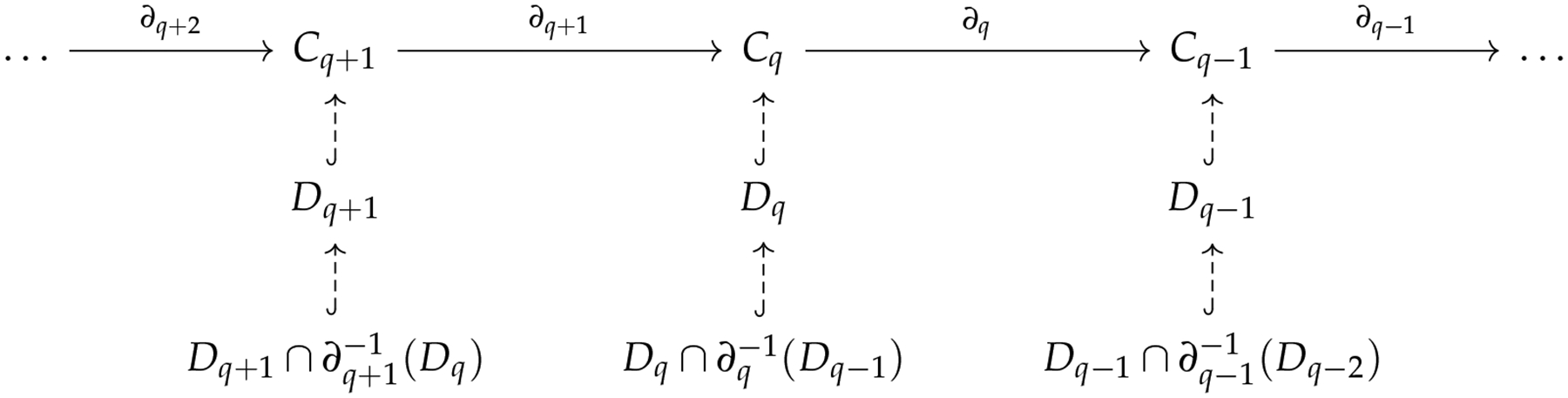
How to costruct embedded homology.

A hypergraph H is a pair (V,E) where E is a subset of the power set of V. An element e∈E consisting of q+1 elements is called a q-hyperedge. To define a chain complex for hypergraphs, the problem is identical to what we encounter in path homology. If we define the q-th chain group as the vector space generated by q-hyperedges, the boundary map is not well defined. One solution is to consider associated simplicial complex (simplicial closure) of a hypergraph [131], that is, the minimal simplicial complex that contains a hypergraph. Another solution inspired by path homology is embedded homology [132]. If we examine the chain complex of the associated simplicial complex, each simplicial chain group $C_{q}$ contains $D_{q}$, the vector space generated by q-hyperedges. We only need to restrict the domain of the simplicial boundary operator to

$${\text{Inf}}_{q}=D_{q}\cap \partial_{q}^{-1}\left(D_{q}\right),$$

and then the boundary operator is well-defined (Figure 13).

A hyperdigraph is a hypergraph in which each hyperedge is ordered (there are other definitions of a hyperdigraph [133,134]), and its embedded homology can be defined analogously [61]. The persistent homology of hypergraphs and hyperdigraphs was studied in [132,135,136]. Persistent hypergraph Laplacians were proposed by Liu et al. [60] and persistent hyperdigraph Laplacians were introduced by Chen et al. [61] Alternative approaches to the homology and Laplacians of hypergraphs include [137–142].

### 4.7. Persistent Dirac Operators

In addition to Laplacians, Dirac operators on chain complexes have also been studied [143–148]. Given a chain complex (V,d)

$$\cdots \overset{d_{3}\;}{\to }V_{2}\overset{d_{2}\;}{\to }V_{1}\overset{d_{1}\;}{\to }V_{0}⟶0$$

where each chain group $V_{q}$ is a finite-dimensional inner product space, the q-th Dirac operator $D_{q}$ is represented by the block matrix

$$\begin{array}{cc} & \;V_{0}\;V_{1}\;V_{2}\;\cdots \;V_{q}\;V_{q+1}\\ \begin{array}{c} V_{0}\\ V_{1}\\ V_{2}\\ \vdots \\ V_{q}\\ V_{q+1} \end{array} & \left(\begin{array}{cccccc} 0 & \left[d_{1}\right] & 0 & \cdots & 0 & 0\\ \left[d_{1}^{\text{*}}\right] & 0 & \left[d_{2}\right] & \cdots & 0 & 0\\ 0 & \left[d_{2}^{\text{*}}\right] & 0 & \cdots & 0 & 0\\ \vdots & \vdots & \vdots & \ddots & \vdots & \vdots \\ 0 & 0 & 0 & \cdots & 0 & \left[d_{q+1}\right]\\ 0 & 0 & 0 & \cdots & \left[d_{q+1}^{\text{*}}\right] & 0 \end{array}\right) \end{array}$$

where [ ] denotes a matrix representation of a linear morphism. Dirac operators are closely related to combinatorial Laplacians. If we think of all combinatorial Laplacians as a single operator $dd^{*}+d^{*}d={\left(d+d^{*}\right)}^{2}$ on V, then the q-th Dirac operator is the restriction of the square root $d+d^{*}$ on $V_{0}⊕\cdots ⊕V_{q+1}$. We can also see this by direct computation. The square of $D_{q}$ is

$$\begin{array}{cc} & \;V_{0}\;V_{1}\;V_{2}\;\cdots \;V_{q}\;V_{q+1}\\ \begin{array}{c} V_{0}\\ V_{1}\\ V_{2}\\ \vdots \\ V_{q}\\ V_{q+1} \end{array} & \left(\begin{array}{cccccc} \left[\Delta_{0}\right] & 0 & 0 & \cdots & 0 & 0\\ 0 & \left[\Delta_{1}\right] & 0 & \cdots & 0 & 0\\ 0 & 0 & \left[\Delta_{2}\right] & \cdots & 0 & 0\\ \vdots & \vdots & \vdots & \ddots & \vdots & \vdots \\ 0 & 0 & 0 & \cdots & \left[\Delta_{q}\right] & 0\\ 0 & 0 & 0 & \cdots & 0 & \left[\Delta_{q+1,-}\right] \end{array}\right) \end{array}$$

where $\Delta_{q}$ is the q-th combinatorial Laplacian. Hence, the square of any eigenvalue λ of a Dirac operator must be an eigenvalue of a combinatorial Laplacian.

Recall that when we define persistent Laplacians, we construct an auxiliary subspace $C_{q+1}^{X,Y}$ of $C_{q+1}(Y)$ and a map $\partial_{q+1}^{X,Y}:C_{q+1}^{X,Y}\to C_{q}(X)$. Since $C_{q}(X)$ is actually a subspace of $C_{q}^{X,Y}$, all $C_{q}^{X,Y}$ and $\partial_{q}^{X,Y}$ constitute an auxiliary chain complex

$$\cdots \overset{\partial_{3}^{X,Y}\;}{\to }C_{2}^{X,Y}\overset{\partial_{2}^{X,Y}\;}{\to }C_{1}^{X,Y}\overset{\partial_{1}^{X,Y}\;}{\to }C_{0}^{X,Y}⟶0.$$

The q-th persistent Dirac operator of simplicial complexes X⊂Y is just the q-th Dirac operator in this auxiliary complex. The square of a persistent Dirac operator is not necessarily a block matrix consisting of persistent Laplacians. It is also possible to extend persistent Dirac operators to other settings, such as path complexes and hypergraphs [146].

### 4.8. Mayer Homology

In classical homology theory, the square of the boundary operator d of a chain complex must be zero $\left(d^{2}=0\right)$. However, this constraint can be relaxed in the so-called Mayer homology theory using an N-chain complex [72]. An N-chain complex is a sequence of abelian groups and group morphisms (V,d) where $d^{N}=0$. In fact, a simplicial complex can give rise to an N-chain complex. Recall that in a simplicial chain complex, the boundary operator is given by

$$\partial \left[v_{a_{0}},\ldots ,v_{a_{q}}\right]=\sum_{i}(-1{)}^{i}\left[v_{a_{0}},\ldots ,{\overset{ˆ}{v}}_{a_{i}},\ldots ,v_{a_{q}}\right].$$

For a prime number N, let $\xi =e^{2\pi \sqrt{-1}/N}$, and we can define a generalized boundary operator d by

$$d\left[v_{a_{0}},\ldots ,v_{a_{q}}\right]=\sum_{i}\xi^{i}\left[v_{a_{0}},\ldots ,{\overset{ˆ}{v}}_{a_{i}},\ldots ,v_{a_{q}}\right]$$

and prove that $d^{N}=0$. Although the N-chain complex is not a chain complex in general, observe that for any positive integer n<N

$$C_{q+N-n}(X;C)\overset{d^{N-n}\;}{\to }C_{q}(X;C)\overset{d^{n}\;}{\to }C_{q-n}(X;C)$$

resembles a part of chain complex. We can define the Mayer homology group $H_{q,n}(X)=\text{ker}\;d^{n}/\text{im}\;d^{N-n}$ [72] and Mayer Laplacians (which can be thought of as $\left(d^{n}+{\left(d^{N-n}\right)}^{*}\right)\left({\left(d^{n}\right)}^{*}+\right.\;\left.d^{N-n}\right))$ analogously [73]. When N=2, an N-chain complex reduces to a chain complex, and a Mayer homology group reduces to a normal homology group. Shen et al. [73] also introduced persistent Mayer homology and persistent Mayer Laplacians on N-chain complexes. Suppose X⊂Y, then we have the following commutative diagram


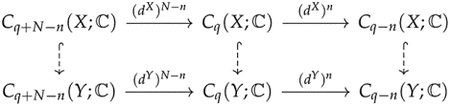




and persistent Mayer Laplacians can be defined analogously. Compared to simplicial homology, Mayer homology and Mayer Laplacians provide more features since we can vary parameters N and n. Mayer homology and the Mayer Laplacians also concern a general relationship between different dimensions. Persistent Mayer homology has been applied to protein-ligand binding affinity predictions [149].

## 5. Conclusions and Outlook

### 5.1. Persistent Topological Laplacians Versus Topological Data Analysis

The development of persistent topological Laplacians (PTLs) was driven by the need to address the limitations of persistent homology in the modeling of complex biomolecular data [24,52]. The kernels of persistent Laplacians have been shown to be isomorphic to their corresponding persistent homology groups, indicating that the information encoded in barcodes is also reflected in the spectra of persistent Laplacians [24,55,56]. Similar theoretical results apply to various PTLs designed for specific data types. The techniques outlined in this survey provide a framework for transforming point clouds or networks into algebraic features that capture both spatial and non-spatial information. These lowdimensional features have proven to be effective in supervised and unsupervised machine learning, uncovering hidden patterns, and demonstrating advantages over classical TDA methods in practical applications [70,71].

Broadly, while traditional TDA has focused predominantly on homology theory, PTLs mark a significant expansion of TDA into spectral theory. Moreover, recent advances, such as the introduction of persistent Dirac operators for flag complexes, digraphs, and hyperdigraphs, open new avenues of exploration [143–148].

The continued extension of TDA into areas such as spectral theory, geometric topology (e.g., persistent Jones polynomial [150] and persistent Khovanov homology [151]), and differential geometry (e.g., persistent de Rham-Hodge theory [52] and persistent Hodge Laplacians [53]) highlights its growing versatility. These developments promise to drive transformative progress in both theoretical research and real-world applications, enhancing the utility of TDA across a wide range of disciplines.

### 5.2. Limitations of Persistent Topological Laplacians

Although persistent topological Laplacians (PTLs) offer significant potential, it is important for researchers to recognize their limitations. The diversity of PTLs, when applied to point clouds [24], differentiable manifolds [52], or 1D curves embedded in higher dimensions [151,152], presents challenges in understanding the intricate relationship between the geometry and topology of the data and the PLT spectra. A deeper understanding of these relationships is essential for the successful and meaningful application of PTLs to real-world problems.

Despite advances in computational algorithms and software development [56,62,63], the computation of PTLs remains computationally intensive, especially for large datasets. Given that the primary strength of topological data analysis (TDA) lies in addressing complex challenges in data science, the development of efficient and robust PTL software packages is among the most pressing needs for advancing the field. Furthermore, the creation of finite field PTLs holds great promise for broadening the applicability of PTLs in data science and beyond.

### 5.3. Future Works

The field of PTLs is dynamic and rapidly evolving. The future development of PTLs is wide open, and we envision the exploration of the following topics:

1. To some extent, the success of TDA can be attributed to its integration with machine learning, particularly with the first introduction of topological deep learning in 2017 [11]. Sheaf neural networks [107], sheaf attention [104], and neural sheaf diffusion [105] are popular topics. Similarly, the development of efficient PTL representations for machine learning, including deep learning, is also an important topic. The featurization of Laplacians typically requires domain knowledge and experience. Since self-learning representations of persistent diagrams have been proposed [153], we wonder if self-learning representations of (persistent) Laplacians are possible. As the eigenvectors of the PTLs were found to have better descriptive power than eigenvalues [57], featurization of the PTL eigenvectors is also an interesting future topic.
2. PTLs have been formulated on a variety of mathematical objects, including simplicial complexes, directed flag complexes, path complexes, cellular sheaves, digraphs, hypergraphs, and hyperdigraphs. One can also extend PTLs to settings such as the Hochschild complex [154], quantum homology [155], multiparameter persistent homology [156], and interaction homotopy and interaction homology [157,158]. We expect that these developments will further extend the scope and capability of the current TDA for real-world applications.
3. It is possible that persistent sheaf Dirac operators can be devised to distinguish certain geometric shapes. Additionally, persistent Dirac operators defined on a spinor bundle may extend persistence to index theory, such as multiscale index theory.
4. The PTLs on manifolds, such as the evolutionary de Rham-Hodge theory, pose implementation challenges compared to their discrete counterparts on point clouds [34]. Recently, the persistent de Rham-Hodge Laplacian in Eulerian representation has been proposed for manifold topological learning (MTL) [53]. Persistent de Rham-Hodge Laplacians extend earlier persistent homology in the cubical setting [159–161]. From a theoretical point of view, it will be interesting to extend various PTLs to the setting of manifolds (with boundaries).
5. In addition to point cloud data and data in manifolds, there are knot-type data, such as DNA packaging in Hi-C data and entangled brain neurons. Knots are traditionally studied with invariants, such as the Alexander polynomial, the Jones polynomial, and the Kauffman polynomial [162]. Song et al. proposed the multiscale Jones polynomial and the persistent Jones polynomial [150]. Khovanov homology [163] is a major breakthrough in knot theory. Shen et al. [151] proposed an evolutionary Khovanov homology for weighted links. Jones and Wei [152] proposed Khovanov Laplacians and showed that, at least for chiral prime knots up to 10 crossings, they can distinguish chiral knots from their mirrors. Based on these developments, PTLs on knot- or curve-type data, i.e., persistent Khovanov Laplacians, can be formulated, and future research on computational geometric topology is widely open.
6. Finally, ChatGPT ushers in a new era of artificial intelligence (AI), offering wide-ranging opportunities in all disciplines. ChatGPT and other chatbots effectively transform pure mathematical theories into practical computational tools, including PTLs [164]. Both AI-enabled topology and topology-enabled AI will have a growing impact on research [165].

## Data Availability

The data will be made available by the authors on request.
